# Drug sensitivity prediction with normal inverse Gaussian shrinkage informed by external data

**DOI:** 10.1002/bimj.201900371

**Published:** 2020-07-23

**Authors:** Magnus M. Münch, Mark A. van de Wiel, Sylvia Richardson, Gwenaël G. R. Leday

**Affiliations:** ^1^ Department of Epidemiology & Biostatistics Amsterdam UMC VU University Amsterdam The Netherlands; ^2^ Mathematical Institute Leiden University Leiden The Netherlands; ^3^ MRC Biostatistics Unit University of Cambridge Cambridge Institute of Public Health Forvie Site, Robinson Way, Cambridge Biomedical Campus Cambridge United Kingdom

**Keywords:** drug sensitivity, empirical Bayes, Genomics of Drug Sensitivity in Cancer (GDSC), variational Bayes

## Abstract

In precision medicine, a common problem is drug sensitivity prediction from cancer tissue cell lines. These types of problems entail modelling multivariate drug responses on high‐dimensional molecular feature sets in typically >1000 cell lines. The dimensions of the problem require specialised models and estimation methods. In addition, external information on both the drugs and the features is often available. We propose to model the drug responses through a linear regression with shrinkage enforced through a normal inverse Gaussian prior. We let the prior depend on the external information, and estimate the model and external information dependence in an empirical‐variational Bayes framework. We demonstrate the usefulness of this model in both a simulated setting and in the publicly available Genomics of Drug Sensitivity in Cancer data.

## 1 INTRODUCTION

Recently, promising results in precision medicine have sparked an interest in cancer drug sensitivity prediction models (Iorio et al., 2016). Typically, these models predict the drug sensitivity for new patients from a set of molecular features. Development of such models is often done in well‐characterised human cancer tissue cell lines. The current paper presents a novel drug sensitivity prediction model and an application to a real drug sensitivity data set.

Development of such models from cell lines has proven to be difficult (see, e.g. the DREAM 7 challenge in Costello et al. (2014)). Difficulties arise, among others, from the dimensions of the problem. Typically, the data contain hundreds of drugs, thousands of cell lines and thousands of molecular features. An example of a large database of drug responses and molecular features is the Genomics of Drug Sensitivity in Cancer (GDSC) data (Yang et al., 2013), which we will further investigate in Section 5. Other examples of such databases include the Cancer Cell Line Encyclopedia (CCLE) (Li et al., 2019) and the US National Cancer Institute 60 human tumour cell line anticancer drug screen (NCI60) (Shoemaker, 2006). The dimensions of these data prohibit the estimation of standard regression models and typically require some form of regularisation.

The GDSC database contains additional information on both the drugs and molecular features, such as the target pathways and developmental stages of the drugs. Additional online repositories may provide extra information such as the molecular weight of the compounds or the publication signatures of the molecular features. In some cases, prior knowledge on the drug efficacies may be available, from previous experiments. We propose to include these possibly beneficial information sources in the estimation of the sensitivity prediction models in a data‐driven manner. More specifically, we estimate a normal inverse Gaussian (NIG) model, where the extent of regularisation is estimated by an adaptive empirical Bayes procedure, guided by the external information.

We are not the first to work on drug sensitivity prediction models. Reviews on the topic are Azuaje (2017) and Ali and Aittokallio (2019). Zhao and Zucknick (2019) and Mai, Rønneberg, Zhao, Zucknick, and Corander (2019) consider a structured penalized multivariate regression approach. Aben, Vis, Michaut, and Wessels (2016) introduce a two‐stage penalized regression model that includes two different types of molecular features. Ammad‐ud din et al. (2016) and Costello et al. (2014) tackle the problem through a multiple kernel learning approach. Our solution allows for the adaptive incorporation of the external information on drugs and features. This is done by pooling information, both across drugs and features. Estimation of the model is through computational feasible variational Bayes approximations, while empirical Bayes estimation of tuning parameters pools information across drugs and features in a data‐driven manner.

The rest of the paper is structured as follows. In Section 2, we introduce our model, the estimation of which is detailed in Section 3. Section 4 describes a simulation study that investigates the estimation of hyperparameters by the proposed method. In Section 5, we analyse the GDSC data, and we end with a discussion in Section 6 on the pros and cons of the proposed method.

## 2 MODEL

### 2.1 Simultaneous equations model

Let $y_{id}$ be the continuous sensitivity measures for cell lines i=1,⋯,n, and drug d=1,⋯,D. We predict sensitivity from molecular features $x_{ij}$, j=1,⋯,p, collected in $x_{i}={\left[\begin{array}{ccc} x_{i1} & \cdots & x_{ip} \end{array}\right]}^{T}$. We assume that both covariates and responses have been centred per drug and regress the drug sensitivities on the molecular features:

(1) $$y_{id}=x_{i}^{T}\beta_{d}+\varepsilon_{id},\;\text{with}\;\varepsilon_{id}∼N\left(0,\sigma_{d}^{2}\right),$$

where the *p*‐dimensional $\beta_{d}={\left[\begin{array}{ccc} \beta_{1d} & \cdots & \beta_{pd} \end{array}\right]}^{T}$ are the drug‐specific omics feature effects. Note that (1) gives rise to a system of D linear regression equations.

The cell lines used in drug response models are often taken from different tissues. In addition, other clinical covariates might be available. To obtain unbiased feature effects, one may wish to account for these. We do so by introducing unpenalized covariates, the $\beta_{jd}$ coefficients of which are endowed with a flat prior. For the sake of clarity, in the following, such unpenalized covariates are omitted. However, the available software allows for their inclusion.

### 2.2 Bayesian prior model

We carry out inference by endowing the parameters with the following priors:

(2a) $$\beta_{jd}|\gamma_{jd}^{2},\tau_{d}^{2},\sigma_{d}^{2}∼N_{p}\left(0,\gamma_{jd}^{2}\tau_{d}^{2}\sigma_{d}^{2}\right),$$

(2b) $$\gamma_{jd}^{2}∼\mathrm{IG}\left(\phi_{jd},\lambda_{\text{feat}}\right),$$

(2c) $$\tau_{d}^{2}∼\mathrm{IG}\left(\chi_{d},\lambda_{\text{drug}}\right),$$

(2d) $$\sigma_{d}^{2}∼1/\sigma_{d}^{3},$$

where IG(ϕ,λ) denotes an inverse Gaussian distribution with mean ϕ and shape λ>0.

In model (2), $\gamma_{jd}^{2}$ in (2b) denotes a local variance component that is supposed to capture local, feature‐specific variation in the model parameters $\beta_{jd}$ in (2a), while the global variance components $\tau_{d}^{2}$ in (2c) capture the drug‐specific, general trend in $\beta_{d}$. Each drug response is endowed with a random error variance $\sigma_{d}^{2}$, distributed according to (2d).

Prior distributions of the form (2) are often referred to as global‐local shrinkage rules (Polson and Scott, 2011), due to the multiplicative separation of the prior variance into a local component $\gamma_{jd}^{2}$ and a global component $\tau_{d}^{2}$. For appropriate local shrinkage in global–local shrinkage models it is important to account for different noise levels $\sigma_{d}^{2}$ by scaling the $\beta_{jd}$ variances accordingly.

**FIGURE 1:**
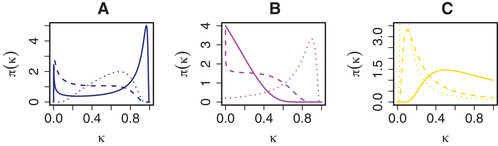
Implied prior densities $\pi (\kappa_{jd})$ for the (A) NIG, (B) Student's *t*, and (C) lasso priors. Different line types correspond to different hyperparameter settings. The hyperparameter settings (given in Section 3 of the SM) were chosen to show some possible, distinct shapes that each of the priors can take

The NIG prior model was introduced in Barndorff‐Nielsen (1978) and since Barndorff‐Nielsen (1997), it is routinely applied in mathematical finance (see, e.g. Kalemanova, Schmid, and Werner (2007)). Here, we extend it with an additional global variance component $\tau_{d}^{2}$. Supplementary material (SM) Section S2 contains more details on the NIG prior. To illustrate the effect of the NIG prior on the posterior mean, we consider the prior reparametrised as in Carvalho, Polson, and Scott (2009), that is, in terms of shrinkage weights $\kappa_{jd}=1/\left(1+\gamma_{jd}^{2}\right)\in \left(0,1\right)$. Under the (simplified) normal means model, that is, $X={\left[\begin{array}{cc} x_{1} & \cdots x_{n} \end{array}\right]}^{T}=I_{p}$, with fixed $\tau_{d}^{2}=\sigma_{d}^{2}=1$, the resulting conditional posterior mean for the $\beta_{jd}$ is $E\left(\beta_{jd}|y_{jd},\kappa_{jd}\right)=\left(1-\kappa_{jd}\right)y_{jd}$. Thus, $\kappa_{jd}=0$ implies no shrinkage of $\beta_{jd}$ and $\kappa_{jd}=1$ implies full shrinkage towards zero. Figure 1 depicts the prior on $\kappa_{jd}$ implied by several choices of $\beta_{jd}$ prior.

Figure 1 shows that, depending on the choice of hyperparameters, the NIG prior can behave similarly to the Student's *t* prior (decreasing form zero, with substantial mass close to zero and little mass close to one, like the solid lines in Figs. 1A,B), but also rather differently (dashed and dotted lines in Figs. 1A,B). Our argumentation to model the $\gamma_{jd}^{2}$ by an inverse Gaussian distribution, as has been suggested in Fabrizi, Greco, and Trivisano (2016) and Caron and Doucet (2008), is three‐fold: (i) the NIG model is more flexible than the lasso prior (as seen from Fig. 1), (ii) the NIG prior allows to model the means of the $\gamma_{jd}^{2}\left(\phi_{jd}\right)$ and $\tau_{jd}^{2}\left(\chi_{d}\right)$ as a function of external data more conveniently than the Student's *t* prior, as explained in Section 3.2 and (iii) like the horseshoe (Carvalho et al., 2009), the NIG shrinkage weights prior can put mass both near zero and one, a desirable property of shrinkage priors (Polson and Scott, 2011).

A few remarks on the choice of error variance prior are justified here: many authors endow error variance components with vague gamma priors. Gelman (2006), among others, advises against this practice. The degree of ‘vagueness’ has a large influence on the posterior, while degree of ‘vagueness’ is a difficult parameter to set. This influence is especially pronounced if the likelihood is relatively flat, as may be reasonably expected in the large *p*, small *n* setting. We therefore model the error variance with Jeffreys objective prior (Jeffreys, 1946) that does not depend on any subjective specification of hyperparameters. In the derivation of our Jeffreys prior for the error variance, we jointly consider an unknown data mean and variance (Kass and Wasserman, 1996). This joint consideration results in the somewhat unorthodox $1/\sigma^{3}$ Jeffreys prior.

### 2.3 External information

In drug sensitivity prediction models, external information on both the drugs and features is often available. Here, we assume this information to be available as external feature ‘covariates’ $c_{jdg}$, for g=1,⋯,G, and drug ‘covariates’ $z_{dh}$, for h=1,⋯,H. An example of a (binary) feature covariate is target pathway presence, with $c_{jdg}=0$ if gene *j* is present in the target pathway of drug *d* and $c_{jdg}=1$ if it is not. An example of a (ternary) drug covariate is developmental phase, with levels experimental phase, clinical development and approved by a governing agency.

The external covariates come in through our mean models for the $\gamma_{jd}^{2}$ and $\tau_{d}^{2}$ hyperpriors: $\phi_{jd}={\left(c_{jd}^{T}\alpha_{\text{feat}}\right)}^{-1}$ and $\chi_{d}={\left(z_{d}^{T}\alpha_{\text{drug}}\right)}^{-1}$, with $c_{jd}=\left[\begin{array}{ccc} c_{jd1} & \cdots & c_{jdG} \end{array}\right]$ and $z_{d}=\left[\begin{array}{ccc} z_{d1} & \cdots & z_{dH} \end{array}\right]$, where categorical external covariates are dummy coded. The model now requires hyperparameters $\alpha_{\text{feat}}$, $\lambda_{\text{feat}}$, $\alpha_{\text{drug}}$ and $\lambda_{\text{drug}}$, which we estimate in a data‐driven manner (see Section 3.2).

**FIGURE 2:**
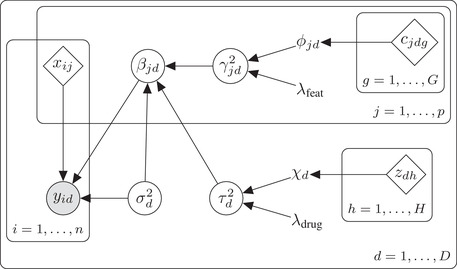
Hierarchical representation of the drug sensitivity prediction model. Grey circles represent observed variables, white circles represent unobserved variables, tilted squares represent fixed data, and unenclosed letters are parameters to be estimated. Cell lines are indexed by *i*, features by *j*, drugs by *d*, drug covariates by *h*, and feature covariates by *g*. The $y_{id}$ are the drug sensitivities, $x_{ij}$ the molecular features, $c_{jdg}$ the external feature covariates, $z_{dh}$ the external drug covariates, $\beta_{jd}$ the regression coefficients, $\sigma_{d}^{2}$ the error variances, $\tau_{d}^{2}$ and $\gamma_{jd}^{2}$ the drug and feature specific variance components, respectively, and $\phi_{jd}$, $\lambda_{\text{feat}}$, $\chi_{d}$, and $\lambda_{\text{drug}}$ the hyperparameters

A representation of our model as a Bayesian DAG is given in Figure 2. We note that in many settings, the set of features might be different for different drugs. In that case the covariates are indexed by the drug *d*: $X^{d}$, a trivial extension of model (1) and (2). This extension is included in the available software, but for clarity it is omitted in the following.

## 3 ESTIMATION

### 3.1 Variational Bayes

The posterior corresponding to the model described in (1) and (2) is not available in closed form. To avoid computationally intensive Markov chain Monte Carlo (MCMC) algorithms, we approximate the joint posterior by variational Bayes (see Blei, Kucukelbir, & McAuliffe, 2017, for a review), where the approximate posterior density factorises as: $p\left(\beta_{d},\gamma_{d}^{2},\tau_{d}^{2},\sigma_{d}^{2}|y_{d}\right)\approx Q_{d}\left(\cdot \right)=q\left(\beta_{d}\right)\cdot q\left(\gamma_{d}^{2}\right)\cdot q\left(\tau_{d}^{2}\right)\cdot q\left(\sigma_{d}^{2}\right)$, where $\gamma_{d}^{2}={\left[\begin{array}{ccc} \gamma_{1d}^{2} & \cdots & \gamma_{pd}^{2} \end{array}\right]}^{T}$. For notational convenience, we slightly abuse notation and let q(·) denote different densities for different inputs. Under such a factorisation, the marginal variational posteriors that minimise the Kullback–Leibler divergence of the true posterior to the variational Bayes approximation (Neal and Hinton, 1998) are given by:

$$\begin{array}{cc} q(\beta_{d}) & \overset{D}{=}N_{p}\left(\mu_{d},\Sigma_{d}\right),\\ q(\gamma_{d}^{2}) & \overset{D}{=}\prod_{j=1}^{p}\mathrm{GIG}\left(-1,\lambda_{\text{feat}}/\phi_{jd}^{2},\delta_{jd}\right),\\ q(\tau_{d}^{2}) & \overset{D}{=}\mathrm{GIG}\left(-\frac{p+1}{2},\lambda_{\text{drug}}/\chi_{d}^{2},\eta_{d}\right),\\ q(\sigma_{d}^{2}) & \overset{D}{=}\Gamma^{-1}\left(\frac{n+p+1}{2},\zeta_{d}\right), \end{array}$$

where GIG(p,ν,η) denotes the generalized inverse Gaussian distribution with index p∈R, and scales ν>0 and η>0 (Jørgensen, 1982). See SM Section S5 for the derivations. The variational parameters $\mu_{d}$, $\Sigma_{d}$, $\delta_{jd}$, $\eta_{d}$ and $\zeta_{d}$ contain cyclic dependencies and are iteratively updated by:

(3a) $$\Sigma_{d}^{\left(h+1\right)}={\left(a_{d}^{\left(h\right)}\right)}^{-1}{\left[X^{T}X+g_{d}^{\left(h\right)}\text{diag}\left(b_{jd}^{\left(h\right)}\right)\right]}^{-1},$$

(3b) $$\mu_{d}^{\left(h+1\right)}={\left[X^{T}X+g_{d}^{\left(h\right)}\text{diag}\left(b_{jd}^{\left(h\right)}\right)\right]}^{-1}X^{T}y_{d},$$

(3c) $$\delta_{jd}^{\left(h+1\right)}=a_{d}^{\left(h\right)}g_{d}^{\left(h\right)}\left[{\left(\mu_{jd}^{\left(h+1\right)}\right)}^{2}+{\left(\Sigma_{d}^{\left(h+1\right)}\right)}_{jj}\right]+\lambda_{\text{feat}},$$

(3d) $$\eta_{d}^{\left(h+1\right)}=a_{d}^{\left(h\right)}\sum_{j=1}^{p}b_{jd}^{\left(h+1\right)}\left[{\left(\mu_{jd}^{\left(h+1\right)}\right)}^{2}+{\left(\Sigma_{d}^{\left(h+1\right)}\right)}_{jj}\right]+\lambda_{\text{drug}},$$

(3e) $$\zeta_{d}^{\left(h+1\right)}=\frac{1}{2}[y_{d}^{T}y_{d}-2y_{d}^{T}X\mu_{d}^{\left(h+1\right)}+\text{tr}\left(X^{T}X\Sigma_{d}^{\left(h+1\right)}\right)+{\left(\mu_{d}^{\left(h+1\right)}\right)}^{T}X^{T}X\mu_{d}^{\left(h+1\right)}$$

(3f) $$+g_{d}^{\left(h+1\right)}\text{tr}\left[\text{diag}\left(b_{jd}^{\left(h+1\right)}\right)\Sigma_{d}^{\left(h+1\right)}\right]+g_{d}^{\left(h+1\right)}{\left(\mu_{d}^{\left(h+1\right)}\right)}^{T}\text{diag}\left(b_{jd}^{\left(h+1\right)}\right)\mu_{d}^{\left(h+1\right)}],$$

until convergence, where $y_{d}={\left[\begin{array}{ccc} y_{1d} & \cdots & y_{nd} \end{array}\right]}^{T}$. Here, we set

(4) $$\begin{array}{cc} a_{d}^{\left(h\right)} & =E_{Q^{\left(h\right)}}(\sigma_{d}^{-2})=\left(n+p+1\right)/\left(2\zeta_{d}^{\left(h\right)}\right),\\ b_{jd}^{\left(h\right)} & =E_{Q^{\left(h\right)}}(\gamma_{jd}^{-2})=\sqrt{\frac{\lambda_{\text{feat}}}{\phi_{jd}^{2}\delta_{jd}^{\left(h\right)}}}\frac{K_{0}\left(\sqrt{\delta_{jd}^{\left(h\right)}\lambda_{\text{feat}}/\phi_{jd}^{2}}\right)}{K_{1}\left(\sqrt{\delta_{jd}^{\left(h\right)}\lambda_{\text{feat}}/\phi_{jd}^{2}}\right)}+\frac{2}{\delta_{jd}^{\left(h\right)}},\\ g_{d}^{\left(h\right)} & =E_{Q^{\left(h\right)}}(\tau_{d}^{-2})=\sqrt{\frac{\lambda_{\text{drug}}}{\chi_{d}^{2}\eta_{d}^{\left(h\right)}}}\frac{K_{(p-1)/2}\left(\sqrt{\eta_{d}^{\left(h\right)}\lambda_{\text{drug}}/\chi_{d}^{2}}\right)}{K_{(p+1)/2}\left(\sqrt{\eta_{d}^{\left(h\right)}\lambda_{\text{drug}}/\chi_{d}^{2}}\right)}+\frac{p+1}{\eta_{d}^{\left(h\right)}}, \end{array}$$

where $K_{\nu }\left(x\right)$ denotes the modified Bessel function of the second kind. A method for fast and numerically stable calculation of ratios of modified Bessel functions of the second kind, as in (4), is given in SM Section S8.

### 3.2 Empirical Bayes

We parametrised the prior mean of the $\gamma_{jd}^{2}$ as $\phi_{jd}={\left(c_{jd}^{T}\alpha_{\text{feat}}\right)}^{-1}$ and the prior mean of $\tau_{d}^{2}$ as $\chi_{d}={\left(z_{d}^{T}\alpha_{\text{drug}}\right)}^{-1}$. This parametrisation allows us to include feature and drug covariates, both continuous and discrete, into the model. Additionally, it reduces the number of hyperparameters from pD to $|\alpha_{\text{feat}}\left|+\right|\alpha_{\text{drug}}|+2$. The Bayesian model then requires the specification of the hyperparameters $\alpha ={\left[\begin{array}{cc} \alpha_{\text{feat}}^{T} & \alpha_{\text{drug}}^{T} \end{array}\right]}^{T}$ and $\lambda ={\left[\begin{array}{cc} \lambda_{\text{feat}} & \lambda_{\text{drug}} \end{array}\right]}^{T}$. These are abstract and hard to interpret parameters for which we generally lack expert knowledge. They do, however, have a significant influence on the shape of the posterior distribution. We therefore propose to estimate these hyperparameters by empirical Bayes. In our case, this results in an objective and data‐driven inclusion of the external feature and drug covariates.

The canonical method for empirical Bayes is to maximise the marginal likelihood with respect to the hyperparameters. In Casella (2001), the marginal likelihood is maximised by an EM algorithm:

$$\begin{array}{cc} \alpha^{\left(l+1\right)},\lambda^{\left(l+1\right)} & =\underset{\alpha ,\lambda >0}{\mathrm{argmax}\;}E_{\cdot |Y}\left[\log p\left(Y,B,\Gamma^{2},\tau^{2},\Sigma^{2}\right)|\alpha^{\left(l\right)},\lambda^{\left(l\right)}\right]\\ & =\underset{\alpha ,\lambda >0}{\mathrm{argmax}\;}E_{\cdot |Y}\left[\log\pi \left(\Gamma^{2}\right)|\alpha_{\text{feat}}^{\left(l\right)},\lambda_{\text{feat}}^{\left(l\right)}\right]+E_{\cdot |Y}\left[\log\pi \left(\tau^{2}\right)|\alpha_{\text{drug}}^{\left(l\right)},\lambda_{\text{drug}}^{\left(l\right)}\right], \end{array}$$

where $Y=\left[\begin{array}{ccc} y_{1} & \cdots & y_{D} \end{array}\right]$, $B=\left[\begin{array}{c} \beta_{1},\cdots ,\beta_{D} \end{array}\right]$, $\tau^{2}={\left[\begin{array}{ccc} \tau_{1}^{2} & \cdots & \tau_{D}^{2} \end{array}\right]}^{T}$, $\Sigma^{2}={\left[\begin{array}{ccc} \sigma_{1}^{2} & \cdots & \sigma_{D}^{2} \end{array}\right]}^{T}$ and $\Gamma^{2}=\left[\begin{array}{ccc} \Gamma_{1}^{2} & \cdots & \Gamma_{D}^{2} \end{array}\right]$, and the expectation is with respect to the joint posterior. In our case, this posterior is not available in closed form, which renders the expectation difficult. While Casella (2001) suggests to approximate the expectation by a Monte Carlo sample, we propose to use the variational Bayes approximation developed in Section 3.1:

$$\alpha^{\left(l+1\right)},\lambda^{\left(l+1\right)}=\underset{\alpha ,\lambda >0}{\mathrm{argmax}\;}E_{Q^{\left(l\right)}}\left[\log\pi \left(\Gamma^{2}\right)|\alpha_{\text{feat}}^{\left(l\right)},\lambda_{\text{feat}}^{\left(l\right)}\right]+E_{Q^{\left(l\right)}}\left[\log\pi \left(\tau^{2}\right)|\alpha_{\text{drug}}^{\left(l\right)},\lambda_{\text{drug}}^{\left(l\right)}\right],$$

where now the expectation is with respect to the converged variational posterior $Q^{\left(l\right)}=\prod_{d=1}^{D}Q_{d}^{\left(l\right)}$. Note that the prior $\Gamma_{d}^{2}$ and $\tau_{d}^{2}$ independence assumption results in separate optimisation problems for the feature hyperparameters ($\alpha_{\text{feat}}$ and $\lambda_{\text{feat}}$), and the drug hyperparameters ($\alpha_{\text{drug}}$ and $\lambda_{\text{drug}}$). If we stack the drug and feature covariates:

$$\begin{array}{c} C=\left[\begin{array}{c} c_{11}^{T}\\ \vdots \\ c_{p1}^{T}\\ \vdots \\ c_{1D}^{T}\\ \vdots \\ c_{pD}^{T} \end{array}\right]\;\text{and}\;Z=\left[\begin{array}{c} z_{1}^{T}\\ \vdots \\ z_{D}^{T} \end{array}\right], \end{array}$$

the empirical Bayes updates are given by:

$$\begin{array}{cc} \alpha_{\text{feat}}^{\left(l+1\right)} & ={\left[C^{T}\text{diag}\left(e_{jd}^{\left(l\right)}\right)C\right]}^{-1}C^{T}1_{pD\times 1},\\ \lambda_{\text{feat}}^{\left(l+1\right)} & =pD{\left[\sum_{d=1}^{D}\sum_{j=1}^{p}b_{jd}^{\left(l\right)}+{\left(\alpha_{\text{feat}}^{\left(l+1\right)}\right)}^{T}C^{T}\text{diag}\left(e_{jd}^{\left(l\right)}\right)C\alpha_{\text{feat}}^{\left(l+1\right)}-2{\left(\alpha_{\text{feat}}^{\left(l+1\right)}\right)}^{T}C^{T}1_{pD\times 1}\right]}^{-1},\\ \alpha_{\text{drug}}^{\left(l+1\right)} & ={\left[Z^{T}\text{diag}\left(f_{d}^{\left(l\right)}\right)Z\right]}^{-1}Z^{T}1_{D\times 1},\\ \lambda_{\text{drug}}^{\left(l+1\right)} & =D{\left[\sum_{d=1}^{D}g_{d}^{\left(l\right)}+{\left(\alpha_{\text{drug}}^{\left(l+1\right)}\right)}^{T}Z^{T}\text{diag}\left(f_{d}^{\left(l\right)}\right)Z\alpha_{\text{drug}}^{\left(l+1\right)}-2{\left(\alpha_{\text{drug}}^{\left(l+1\right)}\right)}^{T}Z^{T}1_{D\times 1}\right]}^{-1}, \end{array}$$

where SM Section S9 shows that $\lambda^{\left(l+1\right)}>0$ and

$$\begin{array}{cc} e_{jd}^{\left(l\right)} & =E_{Q^{\left(l\right)}}\left(\gamma_{jd}^{2}|\alpha_{\text{feat}}^{\left(l\right)},\lambda_{\text{feat}}^{\left(l\right)}\right)=\left(b_{jd}^{\left(l\right)}-2/\delta_{jd}^{\left(l\right)}\right)\cdot \delta_{jd}^{\left(l\right)}{\left(\phi_{jd}^{\left(l\right)}\right)}^{2}/\lambda_{\text{feat}}^{\left(l\right)},\\ f_{d}^{\left(l\right)} & =E_{Q^{\left(l\right)}}\left(\tau_{d}^{2}|\alpha_{\text{drug}}^{\left(l\right)},\lambda_{\text{drug}}^{\left(l\right)}\right)=\left(g_{d}^{\left(l\right)}-\left(p+1\right)/\eta_{d}^{\left(l\right)}\right)\cdot \eta_{d}^{\left(l\right)}{\left(\chi_{d}^{\left(l\right)}\right)}^{2}/\lambda_{\text{drug}}^{\left(l\right)}. \end{array}$$

To ensure proper and unbiased shrinkage, intercepts are included in $\alpha_{\text{feat}}$ and $\alpha_{\text{drug}}$. This is achieved by appending both C and Z with a column of ones. These intercepts are roughly interpreted as the expected prior precisions $E(\gamma_{jd}^{-2})$ and $E(\tau_{d}^{-2})$ if the feature and drug covariates are all zero. Likewise, an α corresponding to an external covariate may be interpreted as an additive effect of the external covariate on the prior expected precision. So an α=1 translates to an increase in expected prior precision of 1 for every increase in the external covariate of 1, keeping all the other external covariates fixed.

Variational Bayes approximations are known to underestimate posterior variances (Rue, Martino, and Chopin, 2009; Consonni and Marin, 2007; Bishop, 2006; Wang and Titterington, 2005). In simulation Scenario 5 in Section S11 of the SM, we compare the variational posterior to MCMC samples from the posterior with fixed hyperparameters estimates (after the procedure described in Section 3.2 has converged). In this simulation scenario and other settings (not shown), the variational approximation to the posterior is accurate. If however, the user is reluctant to trust the variational posterior variances, samples from the posterior may be generated with the Gibbs sampler in SM Section S10. Alternatively, we provide an implementation of the proposed model in stan using the R package rstan (Guo et al., 2018) at https://github.com/magnusmunch/NIG.

## 4 SIMULATIONS

### 4.1 Setup

This section investigates the empirical Bayes estimation properties of the model in a simulated setting; its main aim is to assess hyperparameter estimation. It is a data‐based simulation, wherein the responses are simulated from a synthetic model, but the features are taken from the real GDSC expression data introduced in Section 5. The real GDSC features contain strong collinearities. Such strong collinearities in the design matrix impede correct parameter estimation with small sample sizes. We therefore replace the ambition of correctly estimating the $\beta_{jd}$ with the more modest aim of approximately correct estimation of the hyperparameters.

A pre‐processing step selects 100 features with largest variance, while 251 drug sensitivities for 507 cell lines (half of the total number of cell lines) are simulated from models (1) and (2). We draw the error variances as $\forall d:\sigma_{d}^{2}∼\Gamma^{-1}\left(3,2\right)$, such that the prior $\sigma_{d}^{2}$ mean and variance are both one. We consider the following four scenarios for the simulation of the drug and feature variance components:

- Scenario 1 fixes $\forall d:\tau_{d}^{2}=1$ and draws the $\gamma_{jd}^{2}$ according to model (2). We create four external dummy feature covariates that code for four approximately equally sized groups of features. We set $\alpha_{\text{feat}}$ such that the $\gamma_{jd}^{2}$ of the four groups of features have prior means $\phi_{jd}\in \left\{1,1/2,1/4,1/8\right\}$ ($\alpha_{\text{feat}}={\left[\begin{array}{cccc} 1 & 1 & 3 & 7 \end{array}\right]}^{T}$). The prior scale parameter is set to $\lambda_{\text{feat}}=1$.
- Scenario 2 fixes $\forall j,d:\gamma_{jd}^{2}=1$ and draws the $\tau_{d}^{2}$ according to model (2), following a procedure similar to the procedure for the $\gamma_{jd}^{2}$ in Scenario 1: we create four groups of drugs with corresponding external drug dummy variables and set $\alpha_{\text{drug}}={\left[\begin{array}{cccc} 1 & 1 & 3 & 7 \end{array}\right]}^{T}$, such that we have $\chi_{d}\in \left\{1,1/2,1/4,1/8\right\}$. The scale is set to $\lambda_{\text{drug}}=1$.
- Scenario 3 combines the procedures from Scenarios 1 and 2 to draw both the $\gamma_{jd}^{2}$ and $\tau_{d}^{2}$ according to (2).
- Scenario 4 is equal to Scenario 3, except that we add noise to the external covariates. Noise is supposed to mimic a low external covariate signal and is constructed by permutation of fractions q∈{0.1,0.2,0.33,0.5,0.67,0.8,1} of the rows of the external covariates.

We estimate two models: (i) the NIG model that only includes an intercept in the external covariates, called NIG${}_{f}^{-}$, NIG${}_{d}^{-}$ or NIG${}_{f+d}^{-}$, depending on which variance components are estimated (feature, drug or both in Scenarios 1, 2 and 3/4, respectively), and (ii) the NIG model estimated as in Section 3 that includes all external covariates, called NIG${}_{f}$, NIG${}_{d}$ or NIG${}_{f+d}$, again depending on which variance components are estimated. Exclusion of the external covariates as in the NIG${}_{f}^{-}$, NIG${}_{d}^{-}$ and NIG${}_{f+d}^{-}$ models amounts to direct estimation of common expected prior means ϕ and/or χ, instead of regression estimates for the $\phi_{jd}$ and/or $\chi_{d}$ as in the NIG${}_{f}$, NIG${}_{d}$ and NIG${}_{f+d}$ models. In the language of Polson and Scott (2011) as introduced in Section 1, models NIG${}_{f}$ and NIG${}_{d}$ may be described as local and global shrinkage rules, respectively, as opposed to the global–local shrinkage models NIG${}_{f+d}$ and NIG${}_{f+d}^{-}$. We repeat every simulation Scenario 100 times.

SM Section S11 contains more simulation results for Scenarios 1–4 for the NIG model and the (i) frequentist lasso and (ii) ridge models. Additionally, SM Section S11 contains a comparison of MCMC and VB posteriors in simulation Scenario 3.

### 4.2 Results

**FIGURE 3:**
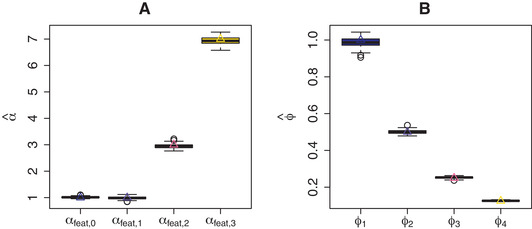
Simulation results for Scenario 1 ($\tau_{d}^{2}$ fixed): estimated and true values for (A) $\alpha_{\text{feat}}$ and (B) prior means $\phi_{jd}$

Figure 3 shows the estimated $\alpha_{\text{feat}}$ together with its true value for NIG${}_{f}$ in Scenario 1 of the simulation study (fixed $\tau_{d}^{2}$). Figure 3A shows that estimation of $\alpha_{\text{feat}}$ is accurate. This results in accurate estimates on the $\phi_{jd}$ scale as well (Fig. 3B). Model NIG${}_{f}^{-}$ (that excludes the external covariates) gives a mean ϕ estimate of 0.457 (0.007) (standard deviation between parentheses), about equal to the true mean of the $\phi_{jd}$, 0.469. Scale $\lambda_{\text{feat}}=1$ is overestimated by NIG${}_{f}$ at 1.321 (0.08), while NIG${}_{f}^{-}$ underestimates at 0.404 (0.013).

**FIGURE 4:**
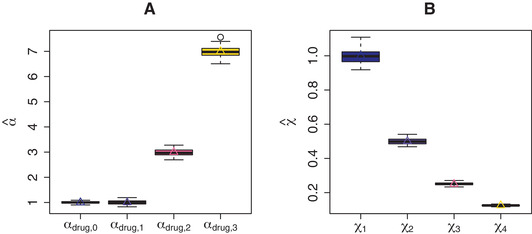
Simulation results for Scenario 2 ($\gamma_{jd}^{2}$ fixed): estimated (boxplots) and true values (triangles) for (A) $\alpha_{\text{feat}}$ and (B) prior means $\phi_{jd}$

Figure 4A shows the accurately estimated $\alpha_{\text{drug}}$ together with the true value for NIG${}_{d}$ in Scenario 2 of the simulation study (fixed $\gamma_{jd}^{2}$). Likewise, the estimates are accurate on the $\chi_{d}$ scale (Fig. 4B). The mean χ estimate in the NIG${}_{d}^{-}$ model is 0.471 (0.012), which is about equal to the true mean 0.469. Scale $\lambda_{\text{drug}}=1$ is overestimated by NIG${}_{d}$ at 12.944 (2.325) and underestimated by NIG${}_{d}^{-}$ at 0.592 (0.023).

**FIGURE 5:**
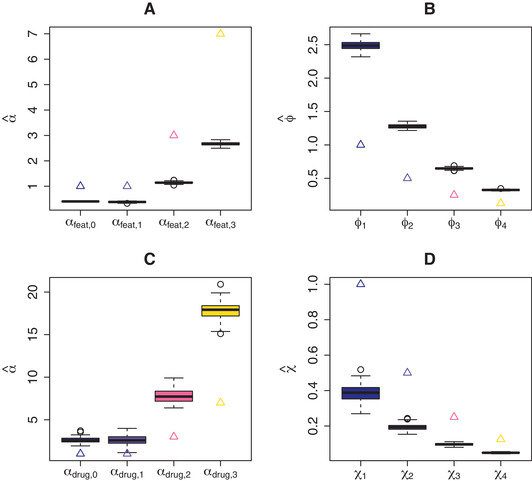
Simulation results for Scenario 3: estimated (boxplots) and true values (triangles) for (A) $\alpha_{\text{feat}}$, (B) prior means $\phi_{jd}$, (C) $\alpha_{\text{drug}}$, and (D) prior means $\chi_{d}$

**FIGURE 6:**
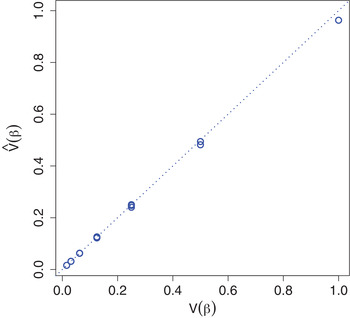
Simulation results for Scenario 3: mean estimated prior variances $V(\beta_{jd})$ versus true values, with line of identity (dotted)

In Figure 5 the $\alpha_{\text{feat}}$ and $\alpha_{\text{drug}}$ estimated by NIG${}_{f+d}$ are displayed together with their true values for simulation Scenario 3. $\alpha_{\text{feat}}$ are underestimated (Fig. 5A), while $\alpha_{\text{drug}}$ (Fig. 5C) are overestimated, resulting in overestimated $\phi_{jd}$ (Fig. 5B) and underestimated $\chi_{d}$ (Fig. 5D), respectively. The biases seem to be consistent though. The mean ratios ϕ_1_ to ϕ_2_, ϕ_3_, ϕ_4_ are 0.514 (0.017), 0.26 (0.009), 0.131 (0.005), while the mean ratios χ_1_ to χ_2_, χ_3_, χ_4_ are 0.507 (0.078), 0.253 (0.036), 0.128 (0.017). In both cases the true values are 0.5, 0.25, 0.125, so in a relative sense, the $\alpha_{\text{feat}}$ and $\alpha_{\text{drug}}$ estimates are about correct. Moreover, overestimation of the $\phi_{jd}$ is compensated for by the underestimation of the $\chi_{d}$: the estimated mean prior variances $V\left(\beta_{jd}\right)=\phi_{jd}\cdot \chi_{d}$ (ignoring the error variance) are unbiased (Fig. 6). The NIG${}_{f+d}^{-}$ model is also consistently over‐ and underestimating ϕ and χ with mean estimates 0.971 (0.018) and 0.207 (0.013), respectively (compared to the true mean 0.469). Again, on the $V(\beta_{jd})$ level, this bias almost vanishes; the mean estimated $V(\beta_{jd})$ (ignoring error variance) are 0.201 (0.014) while their true mean is 0.22. In Scenario 3, NIG${}_{f+d}$ overestimates $\lambda_{\text{feat}}=1$ at 7.098 (0.621) and underestimates $\lambda_{\text{drug}}=1$ at 0.437 (0.047). Similar results hold for NIG${}_{f+d}^{-}$ with $\lambda_{\text{feat}}$ estimate 1.5 (0.066) and $\lambda_{\text{drug}}$ estimate 0.195 (0.014).

**FIGURE 7:**
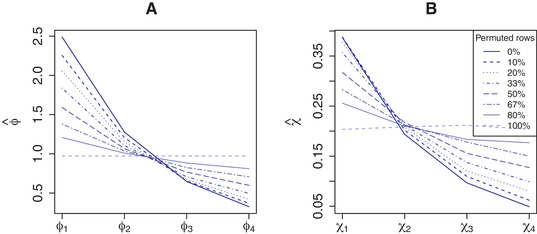
Simulation results for Scenario 3: mean estimated prior means (A) $\phi_{jd}$, and (B) $\chi_{d}$ for different levels of noise in the external covariates

Figure 7 displays the mean $\phi_{jd}$ and $\chi_{d}$ estimates for different noise levels in simulation Scenario 4. The simulation shows that with increasing noise level, the estimated prior means $\phi_{jd}$ and $\chi_{d}$ for the four groups of external covariates become more and more alike. In other words, noise in the external covariates impedes estimation of $\alpha_{\text{feat}}$ and $\alpha_{\text{drug}}$, as expected.

**FIGURE 8:**
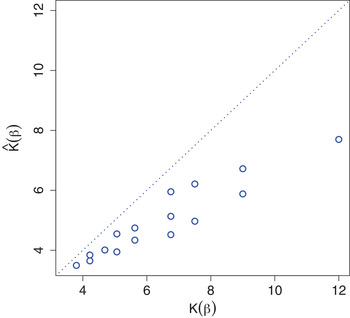
Simulation results for Scenario 3: mean estimated prior Kurtoses $K(\beta_{jd})$ versus true values, with line of identity (dotted)

To summarise, estimation of only $\alpha_{\text{feat}}$ or $\alpha_{\text{drug}}$ (and consequently $\phi_{jd}$ and $\chi_{d}$) by NIG${}_{f}$ and NIG${}_{d}$, respectively is relatively unbiased, as evident from simulation Scenarios 1 and 2 (Figs. 3 and 4). In contrast, simultaneous estimation in Scenario 3 results in overestimated $\phi_{jd}$ and underestimated $\chi_{d}$ by NIG${}_{f+d}$ (Fig. 5). We conjecture that this interplay of drug and feature variance components is due to near‐unidentifiability. In any case, the consequences are limited, since the variances on the $\beta_{jd}$ level are left unbiased (Fig. 6). If separately estimated, scale parameters $\lambda_{\text{feat}}$ and $\lambda_{\text{drug}}$ are overestimated by NIG${}_{f}$ and NIG${}_{d}$, respectively. If estimated simultaneously NIG${}_{f+d}$ overestimates $\lambda_{\text{feat}}$ and underestimated $\lambda_{\text{drug}}$. On the $\beta_{jd}$ level, λ influences kurtoses $K(\beta_{jd})$. Figure 8 shows the mean estimated kurtoses versus their true values. Kurtoses seem to be underestimated by NIG${}_{f+d}$. In contrast, NIG${}_{f+d}^{-1}$ overestimates the true mean of the $K(\beta_{jd})$, 6.4716797, at 10.234 (3.696). Lastly, the results from Scenario 3 in SM Section S11 show that the VB approximation is quite good as compared to standard MCMC.

## 5 GDSC DATA

### 5.1 Primary data

The GDSC project's (Yang et al., 2013) aim is ‘to improve cancer treatments by discovering therapeutic biomarkers that can be used to identify patients most likely to respond to anticancer drug’. Part of the project is to screen >1000 human cancer cell lines for drug sensitivities. The cell lines have been genetically characterised and several drug sensitivity measures are recorded. The data is freely available from Garnett et al. (2012) and consist of: (i) the sensitivity measures of the cell lines to the drugs, (ii) annotation of the screened compounds and (iii) the cell lines' genomic profile (mutations, copy numbers, methylation profiles and gene expression). We will attempt to predict drug sensitivities of the cell lines, as quantified by half maximal inhibitory concentration (IC50), using the gene expression and gene mutation data. Other choices of sensitivity measures than IC50 are possible, but a discussion on the pros and cons of different sensitivity measures is beyond the aim of this paper. We have used the version of the data that is presented in Iorio et al. (2016). We averaged repeated measures over cell line‐drug combinations and model the logarithm of the IC50 values. In the following, IC50 refers to these log‐transformed values. After removing all cell lines with missing values, we end up with 388 to 1043 IC50 estimates for 251 drugs. Differences in the number of cell lines between drugs occur, because not all drug and cell line combinations are available. The pre‐processed expression and mutation data consist of 17,737 and 300 genes, respectively.

### 5.2 External data

Two ternary drug covariates are available: the developmental stage (experimental, in clinical development or clinically approved) of the drugs and the action (unknown, cytotoxic or targeted) of the drugs. These drug covariates are taken directly from the GDSC database's annotation file and dummy coded with reference categories clinically approved drugs and cytotoxic drugs. We expect that drugs that have been clinically approved are easiest to predict and hence yield the largest prior $\beta_{jd}$ variances, followed by the drugs in clinical development, and the experimental drugs. Likewise, we expect the targeted drugs to yield the largest prior $\beta_{jd}$ variances, followed by the cytotoxic drugs, and the unknown target drugs. Note that large $\beta_{jd}$ variances translate to large prior $\gamma_{jd}^{2}$ and $\tau_{d}^{2}$ means.

Furthermore, we have a binary feature covariate available that indicates whether a gene belongs to the drug target pathway. The feature covariate was created by comparing the target pathways in the GDSC annotation to the KEGG (Kanehisa and Goto, 2000) and reactome (Fabregat et al., 2018) repositories. The reference category here is features that are not in the target pathway. For this external covariate, we expect that genes that are in the pathway of the drug are more predictive than genes that are not, that is, they have larger prior $\beta_{jd}$ variances than drugs that are not in the pathway.

The type of molecular marker may be included as external covariate, that is, whether the feature is a gene expression or gene mutation. As an alternative to direct inclusion of the mutation data, we use *p*‐values from the mutations as external covariate. These were obtained from a *t*‐test comparing IC50 values of mutated and unmutated genes. We expect that lower mutation *p*‐values result in a larger prior $\beta_{jd}$ variances.

Lastly, *p*‐values from an analysis of the CCLE data (Li et al., 2019), a database similar to the GDSC, are included as external covariate. These *p*‐values are obtained from a simple correlation between the IC50 values and the gene expressions from the CCLE data. The harmonic mean per gene is then used as external covariate for the GDSC data analysis. Again, we expect a positive relation between these external *p*‐values and the larger prior $\beta_{jd}$ variances.

### 5.3 Analyses

Four analyses were conducted:

- Analysis 1 includes gene expressions as predictors and *p*‐values from the gene mutation data as external covariate (G=1, H=0). A pre‐processing step selects between 221 and 280 genes per drug for which both the expression as well as a mutation *p*‐value is available.
- Analysis 2 includes both gene expressions and mutations as predictors with the feature type as external covariate, that is, wether the feature is a expression or mutation (G=1, H=0). For this analysis, we pre‐select 300 gene expressions with maximum variance and 295 gene mutations for which there are both mutated and wild‐type cell lines available.
- Analysis 3 uses 500 gene expressions as features, selected based on maximum variance. The CCLE *p*‐values are included as external covariates (G=1, H=0).
- Analysis 4 includes the gene expressions as features and both the annoted drug variables and pathway status of the genes as external covariates (G=1, H=2, before dummy coding). We pre‐select 500 genes expressions based on maximum variance.

In all analyses we estimated the same models as in the simulations (Section 4 and SM Section S11): (i) NIG${}_{f+d}^{-}$, (ii) NIG${}_{f+d}$ and (iii) frequentist lasso and (iv) ridge models.

In all analyses we use all cell lines to estimate the hyperparameters presented in Section 5.4. Mean prediction mean squared errors (PMSE) and its standard error are estimated by 10‐fold cross validation, where $\text{PMSE}=D^{-1}n^{-1}\sum_{d=1}^{D}\sum_{i=1}^{n}{\left(y_{d}-x_{i}^{T}\hat{\beta_{d}}\right)}^{2}$, with $\hat{\beta_{d}}$ the estimator for $\beta_{d}$. In the NIG model, that provides full posteriors, the posterior mean $E(\beta_{d}|y_{d})$ is used as point estimate.

### 5.4 Results

The non‐zero NIG${}_{f+d}$ α estimates in Tables 1–5 show that there is an effect of the external covariates.

- Analysis 1 results in a positive additive effect of the mutation *p*‐values on the prior $\beta_{d}$ precisions (Table 1). This translates to more $\beta_{d}$ shrinkage towards zero with increasing *p*‐value, as expected.
- Analysis 2 shows that gene mutations are more predictive than gene expressions, as observed from the negative effect of mutation dummy on prior precisions (Table 2): mutations are shrunken less than expressions.
- Analysis 3 indicates that CCLE *p*‐values are positively related to prior precision (Table 3), that is, higher CCLE *p*‐values results in more shrinkage of $\beta_{d}$, as expected.
- Analysis 4 gives a negative effect for the pathway dummy (Table 4), indicating less shrinkage for genes that are in the drugs' target pathway, as expected. According to expectation, experimental and developmental drugs prior precisions are shrunken more than the reference category, approved drugs (Table 5). Somewhat surprisingly, targeted and unknown drugs are shrunken more than the reference, cytotoxic drugs (Table 5), although the differences on the $\beta_{d}$ variance scale are small: $\hat{E}\left(\tau_{d}^{2}\right)=\left\{0.0035,0.0034,0.0031\right\}$ for cytotoxic, targeted, and unknown drugs, respectively (ignoring other variance components).

**TABLE 1:** α estimates from analysis 1

	Feature intercept	*p*‐value	Drug intercept
NIG${}_{f+d}^{-}$	2.616		280.408
NIG${}_{f+d}$	2.551	0.179	282.068

*Note*. Empty cells correspond to fixed zero parameters.

**TABLE 2:** α estimates from analysis 2

	Feature intercept	Mutation	Drug intercept
NIG${}_{f+d}^{-}$	3.025		171.623
NIG${}_{f+d}$	3.884	−1.781	153.217

*Note*. Empty cells correspond to fixed zero parameters.

**TABLE 3:** α estimates from analysis 3

	Feature intercept	*p*‐value	Drug intercept
NIG${}_{f+d}^{-}$	2.84		330.614
NIG${}_{f+d}$	2.55	0.658	337.086

*Note*. Empty cells correspond to fixed zero parameters.

**TABLE 4:** α estimates from analysis 4

	Feature intercept	Pathway
NIG${}_{f+d}^{-}$	2.841	
NIG${}_{f+d}$	2.845	−0.261

*Note*. Empty cells correspond to fixed zero parameters.

**TABLE 5:** α estimates from analysis 4

	Drug intercept	Experimental	Development	Targeted	Unknown
NIG${}_{f+d}^{-}$	331.403				
NIG${}_{f+d}$	289.290	43.985	53.864	2.845	34.964

*Note*. Empty cells correspond to fixed zero parameters *(continued)*.

The mean PMSE, calculated on the test data, are displayed in Table 6. Note that due to standardisation, an empty reference model has a PMSE of one. In terms of PMSE, ridge outperforms the other models in all Analyses 1, 3 and 4, while NIG${}_{f+d}$ α performs best in Analysis 2. In general however, all models perform very similarly. The ranges of mean PMSE over all methods are 0.0146, 0.0179, 0.0163 and 0.0163, for Analyses 1–4, respectively, indicating that the differences in predictive performance are very small. Furthermore, the difference between NIG${}_{f+d}$, that includes external covariates, and NIG${}_{f+d}^{-}$, that excludes the external covariates is small; an indication that the external covariates are not very informative here.

**TABLE 6:** Mean (standard deviation) of cross‐validated PMSE for GDSC data

	Analysis 1	Analysis 2	Analysis 3	Analysis 4
NIG${}_{f+d}^{-}$	0.796 (0.006)	0.805 (0.007)	0.783 (0.005)	0.785 (0.006)
NIG${}_{f+d}$	0.796 (0.006)	**0.803 (0.007)**	0.783 (0.005)	0.785 (0.006)
ridge	**0.795 (0.005)**	0.807 (0.005)	**0.782 (0.005)**	**0.783 (0.005)**
lasso	0.809 (0.005)	0.821 (0.005)	0.798 (0.005)	0.799 (0.006)

*Note*. Best performing model (per analysis) in bold.

Part of the differences in performance between NIG and ridge may be explained with the different levels of sparsity in the solution. Although NIG does not automatically select features, as opposed to the lasso, its $\beta_{d}$ prior has larger kurtosis than the ridge prior (see SM Section S2). The resulting heavy‐tailedness as compared to the ridge prior facilitates features selection. Selection of features from a sparse prior may be achieved through the decoupling shrinkage and selection approach (DSS) introduced in Hahn and Carvalho (2015). We have applied DSS in our analyses, where we select either (approximately) the same number of features as lasso (about 50 in all analyses), 25 or 100 features. Table 7 compares the resulting mean PMSE values. The table shows that NIG${}_{f+d}$ plus DSS outperforms lasso, for all three numbers of selected features, in all analyses.

**TABLE 7:** Mean (standard deviation) of cross‐validated PMSE for selection methods (number of selected features between parentheses) on GDSC data

	Analysis 1	Analysis 2	Analysis 3	Analysis 4
lasso (25)	0.828 (0.004)	0.823 (0.005)	0.812 (0.005)	0.812 (0.005)
lasso${}_{\lambda^{\text{CV}}}\;\left(∼50\right)$ a	0.809 (0.005)	0.821 (0.005)	0.798 (0.005)	0.799 (0.006)
lasso (100)	0.826 (0.008)	0.841 (0.007)	0.809 (0.006)	0.81 (0.007)
NIG${}_{f+d}+$DSS (25)	0.826 (0.004)	0.814 (0.005)	0.809 (0.005)	0.811 (0.005)
NIG${}_{f+d}+$DSS (∼50)^a^	0.806 (0.004)	0.808 (0.006)	0.792 (0.005)	0.794 (0.006)
NIG${}_{f+d}+$DSS (100)	**0.798 (0.006)**	**0.799 (0.006)**	**0.786 (0.005)**	**0.787 (0.006)**

*Note*. Best performing model (per analysis) in bold.

a Lasso with cross validated λ selects, on average, 42–56 features in all analyses (indicated with ∼50).

To assess model fit, Section S12 in the SM displays the conditional predictive ordinates (CPO) for the NIG${}_{f+d}$ model for the four analyses. A visual inspection of the CPOs learns that no extreme outliers occur.

## 6 DISCUSSION

The preceding presents a novel model for drug sensitivity prediction from a set of high‐dimensional molecular features. The model allows for the inclusion of discrete and continuous external covariates on both the drugs and features. Inclusion of the external information is through data‐driven and adaptive empirical Bayes estimation of the hyperparameters in the NIG prior model (2). Variational Bayes estimation is efficient and scales well with the number of features and samples. Estimation of NIG${}_{f+d}$ in the GDSC data analayses in Section 5 took 20, 24, 17 and 125 min on a 2016 MacBook Pro with 2 GHz Dual‐Core Intel Core i5 processor and 8 GB of memory, running macOS 10.15.1.

Simulation Scenarios 1 and 2 in Section 4 show that estimation of drug‐ and feature‐specific hyperparameters is, in principle, fairly accurate. However, when estimated jointly, biases may occur due to the interplay between the two sources of information. Fortunately, these biases cancel out on the $\beta_{jd}$ level, such that the prior variance estimates $V(\beta_{jd})$ are accurate. Ultimately, predictive performance benefits from the inclusion of external covariates, according to the results in Section 11 of the SM.

The model is put into practice on the GDSC data in Section 5. The comparison of NIG${}_{f+d}$ to NIG${}_{f+d}^{-}$ (that excludes the external covariates) shows that although the inclusion of external covariates substantially modifies the hyperparameters, predictive performance as measured by PMSE is only slightly better in one of four analyses. The NIG model is competitive with convential, penalized methods like lasso and ridge, but all three methods achieve PMSE of only 0.80 in all analyses, a 20% reduction compared to the empty model. In three of the four analyses, ridge slightly outperforms NIG, which in turn outperforms lasso. In Analysis 2, NIG slightly outperforms ridge and lasso. The indications of the above are two‐fold: (i) the GDSC data do not contain a lot of signal overall and (ii) the external covariates are not very informative for the GDSC data. We note however, that NIG seems to have a small advantage in terms of feature selection. If we follow the DSS approach in Hahn and Carvalho (2015) for feature selection, PMSE after selection is slightly better than lasso in all four analyses.

The penalized regression methods estimate penalty parameters by cross‐validation. Cross‐validation directly minimises the (approximate) PMSE, as opposed to empirical Bayes in the NIG that maximises the (approximate) marginal likelihood, a measure of model fit. To achieve maximal predictive accuracy, direct prediction error optimsation by cross‐validation is preferred. However, direct prediction error optimisation by cross‐validation is not feasible in the external covariates setting, due to the large number of hyperparameters. A caveat with penalized regression methods is that they do not give measures of parameter uncertainty. NIG, on the other hand, gives the full posterior of the parameters, either through a variational Bayes approximation or with the Gibbs sampler from SM Section S10. The full posterior gives direct access to the parameter uncertainties for a better interpretable model. In addition, given that the linear predictor is a linear combination of $\beta_{jd}$, and we have access to the approximate multivariate posterior of $\beta_{d}$, NIG also allows to assess uncertainty of the predictions.

An alternative strategy to include external covariates is the varying coefficient (VC) model (Hastie and Tibshirani, 1993). The VC model treats the mean of the regression coefficients as a deterministic function of external covariates, as opposed to our probabilistic model for the variance of the regression coefficients. Ni, Stingo, Ha, Akbani, and Baladandayuthapani (2019) introduces a Bayesian VC model where the relation between the regression coefficients and external covariates is no longer deterministic, but still based on the mean of the coefficients. Besides our computationally more feasible VB‐EM estimation, we advocate for a more indirect model for the relation between external covariates and regression coefficients, that is, through their random variance components. This indirect model assumes less structure about the relation between external covariates and regression coefficients than the direct VC mean model approach. In particular, a VC mean model describes both magnitude and direction of the external covariates effects, while our variance model only describes the magnitude and is invariant to the direction of the effects. Nonetheless, a combination of the two approaches (both mean and variance modelled as functions of the external covariates) may be a fruitful future research direction. Other methods that consider an external covariate model for the variance components of the regression coefficients are bSEM (Leday et al., 2017; Kpogbezan, Vaart, Wieringen, Leday, and van de Wiel, 2017; and xtune (Zeng, Thomas, and Lewinger, 2020). Here, bSEM is designed to include only dichotomous external covariates, so is not applicable in most of the applications and simulations that we have considered here. Inclusion of multiple external features is allowed by xtune, but not on the drug level, so xtune has limited applicability in our simulations and applications.

A possible criticism of the NIG model is the treatment of α as fixed hyperparameters instead of random. A Bayesian could argue that endowment of α with a hyperprior results in propagation of uncertainty about α and as a result improved regression parameter uncertainty quantification. Van de Wiel, te Beest, and Münch (2019) show in a similar setting that EB estimation of hyperparameters does not necessarily lead to worse uncertainty quantification as measured by frequentist coverage of Bayesian credible intervals, as compared to a full Bayes treatment of the hyperparameters.

Possible directions of future research are applications of the NIG model to different data types. Suitable applications are eQTL studies, in which gene expressions are regressed on SNPs. Several interesting external covariates are available, both on the genes as well as the SNPs. An example of such an external covariate for the genes is gene length, where we suspect that longer genes are harder to predict. The distance of the SNP to the gene is an example of an external SNP covariate, where the expectation is that SNPs further from the gene are less predictive of that gene's expression.

## CONFLICT OF INTEREST

The authors have declared no conflict of interest.

### OPEN RESEARCH BADGES

This article has earned an Open Data badge for making publicly available the digitally‐shareable data necessary to reproduce the reported results. The data is available in the Supporting Information section.

This article has earned an open data badge “**Reproducible Research**” for making publicly available the code necessary to reproduce the reported results. The results reported in this article could fully be reproduced.

## Supporting information

Supporting Information

Supporting Information
