# Supplementary material for: Drug sensitivity prediction with normal inverse Gaussian shrinkage informed by external data
Source: Biom J. 2020 Jul 23;63(2):289–304. doi: 10.1002/bimj.201900371 (PMC7891636; doi:10.1002/bimj.201900371)
Supplement: Supplementary file 1 — Supporting Information [file BIMJ-63-289-s002.pdf]

Drug sensitivity prediction with normal inverse Gaussian shrinkage informed by external data

Magnus M. Münch[1,2]*, Mark A. van de Wiel[1,3], Sylvia Richardson[3], and Gwenaël G. R. Leday[3]

April 30, 2020

1. Department of Epidemiology & Biostatistics, Amsterdam UMC, VU University, PO Box 7057, 1007 MB Amsterdam, The Netherlands
2. Mathematical Institute, Leiden University, Leiden, The Netherlands
3. MRC Biostatistics Unit, Cambridge Institute of Public Health, Cambridge, United Kingdom

1 Content overview

This document contains the Supplementary Material (SM) to the document 'Drug sensitivity prediction with normal inverse Gaussian shrinkage informed by external data'. In the following, this document is referred to as Main Document (MD).

Section 2 describes the moments of the normal inverse Gaussian (NIG) prior. Section 3 describes the hyperparameter settings to generate Figure 1 in the MD and Section 4 outlines the derivation to arrive at the Jeffreys prior for the error variance in equation (2d) of the MD. Section 5 gives the variational Bayes (BV) derivations that lead to the estimating equations in the MD, Section 6 gives the evidence lower bound (ELBO) that is maximised during the VB iterations. In Section 7 efficient VB calculations are given, while Section 8 outlines a computationally stable method to calculate ratios of modified Bessel functions. Section 9 shows that the empirical Bayes (EB) scale estimates $\boldsymbol{\lambda}$ are non-negative. Section 10 introduces a Gibbs sampling scheme to sample from the posterior corresponding to the full Bayesian version of the NIG model. Finally, Sections 11 and 12 contain additional results to the simulations and GDSC data analyses in Sections 4 and 5 in the MD, respectively.

2 Normal inverse Gaussian prior moments

Let $\beta \sim \mathcal{N}(0, \sigma^2\tau^2\gamma^2)$, where σ^2 and τ^2 are fixed and $\gamma^2 \sim \mathcal{IG}(\phi, \lambda_{\text{feat}})$, then

$$\begin{aligned}\mathbb{E}(\beta|\sigma^2,\tau^2) &= 0,\\ \mathbb{V}(\beta|\sigma^2,\tau^2) &= \sigma^2\tau^2\phi,\\ \mathcal{K}(\beta|\sigma^2,\tau^2) &= \frac{3\phi}{\lambda_{\text{feat}}} + 3,\end{aligned}$$

*Correspondence to: m.munch@amsterdamumc.nl

where $\mathcal{K}(\cdot)$ denotes the kurtosis. Now letting $\tau^2 \sim \mathcal{IG}(\chi, \lambda_{\text{drug}})$ and integrating it out we have:

$$
\begin{aligned}
&\mathbb{E}(\beta|\sigma^2) = 0, \\
&\mathbb{V}(\beta|\sigma^2) = \sigma^2 \chi \phi, \\
&\mathcal{K}(\beta|\sigma^2) = 3 \left(\frac{\phi}{\lambda_{\text{feat}}} \frac{\chi}{\lambda_{\text{drug}}} + \frac{\phi}{\lambda_{\text{feat}}} + \frac{\chi}{\lambda_{\text{drug}}} \right) + 3.
\end{aligned}
$$

3 Hyperparameter settings

For the NIG shrinkage weights prior in Figure 1a the following hyperparameter settings (in terms of the inverse Gaussian prior) were used: $\lambda = 1$, $\phi = 10$ (solid), $\lambda = 0.1$, $\phi = 10$ (dashed), and $\lambda = 1$, $\phi = 1$ (dotted). The Student's t prior on the regression parameters β_{jd} arises if $\gamma_{jd}^2 \sim \Gamma^{-1}(a, b)$, with $\Gamma^{-1}(a, b)$ an inverse Gamma distribution with shape a and scale b. Figure 1b is then obtained by setting: $a = 0.9$, $b = 1.3$ (solid), $a = 1$, $b = 4$ (dashed), and $a = 1$, $b = 0.2$ (dotted). The lasso prior results from an exponential prior on γ_{jd}^2. To create Figure 1c the following settings were used for exponential rate λ: $\lambda = 1$ (solid), $\lambda = 0.2$ (dashed), and $\lambda = 0.1$ (dotted).

4 Jeffrey's prior

We parametrise the data with a Gaussian distribution with unknown mean and variance, i.e., $y_i \sim \mathcal{N}(\mu, \sigma^2)$. This results in the following Hessian (matrix of second derivatives) of the log density:

$$
\mathbf{H}(\log f(y_i|\mu, \sigma^2)) = \begin{bmatrix} -\frac{1}{\sigma^2} & y_i - \mu \\ y_i - \mu & \frac{1}{2\sigma^4} - \frac{(y_i - \mu)^2}{\sigma^6}. \end{bmatrix}
$$

Jeffreys prior (Jeffreys, 1946) is now found as:

$$
\pi(\mu, \sigma^2) \propto |\mathcal{I}(\mu, \sigma^2)|^{1/2} = |-\mathbb{E}(\mathbf{H})|^{1/2} = \begin{vmatrix} \frac{1}{\sigma^2} & 0 \\ 0 & \frac{1}{2\sigma^4} \end{vmatrix}^{1/2} \propto 1/\sigma^3,
$$

where $\mathcal{I}(\theta)$ denotes the Fisher information of θ.

5 Variational Bayes derivations

For clarity we have indexed the variational density functions with their respective parameters, which we omitted in the MD. In the following all expectations are with respect to the variational posterior Q_d. The variational posterior for $\boldsymbol{\beta}_d$ is found as follows:

$$
\begin{aligned}
\log q_{\boldsymbol{\beta}_d}(\boldsymbol{\beta}_d) &\propto \mathbb{E}[\log \mathcal{L}(\mathbf{y}_d|\boldsymbol{\beta}_d, \sigma_d^2)] + \mathbb{E}[\log \pi(\boldsymbol{\beta}_d|\boldsymbol{\gamma}_d^2, \sigma_d^2, \tau_d^2)] \\
&\propto -\frac{1}{2}\sum_{i=1}^n \mathbb{E}\left[\frac{(y_{id} - \mathbf{x}_i^{\mathrm{T}}\boldsymbol{\beta}_d)^2}{\sigma_d^2}\right] - \frac{1}{2}\sum_{j=1}^p \mathbb{E}\left(\frac{\beta_{jd}^2}{\gamma_{jd}^2\sigma_d^2\tau_d^2}\right), \\
q_{\boldsymbol{\beta}_d}(\boldsymbol{\beta}_d) &\overset{D}{=} \mathcal{N}_p(\boldsymbol{\mu}_d, \boldsymbol{\Sigma}_d), \\
&\quad \text{with } \boldsymbol{\Sigma}_d = \mathbb{E}(\sigma_d^{-2})^{-1}\{\mathbf{X}^{\mathrm{T}}\mathbf{X} + \mathbb{E}(\tau_d^{-2})\text{diag}[\mathbb{E}(\gamma_{jd}^{-2})]\}^{-1}, \\
&\quad \text{and } \boldsymbol{\mu}_d = \{\mathbf{X}^{\mathrm{T}}\mathbf{X} + \mathbb{E}(\tau_d^{-2})\text{diag}[\mathbb{E}(\gamma_{jd}^{-2})]\}^{-1}\mathbf{X}^{\mathrm{T}}\mathbf{y}_d.
\end{aligned}
$$

The variational posterior for γ_{jd}^2 is given by:

$$\begin{aligned}
\log q_{\gamma_{jd}^2}(\gamma_{jd}^2) &\propto \mathbb{E}[\log \pi(\beta_{jd}|\gamma_{jd}^2, \sigma_d^2, \tau_d^2)] + \log \pi(\gamma_{jd}^2) \\
&\propto -\frac{1}{2}\log \gamma_{jd}^2 - \frac{1}{2}\mathbb{E}\left(\frac{\beta_{jd}^2}{\sigma_d^2 \tau_d^2}\right)\gamma_{jd}^{-2} - \frac{3}{2}\log \gamma_{jd}^2 - \frac{\lambda_{\text{feat}}\{\gamma_{jd}^2 - \phi_{jd}\}^2}{2\phi_{jd}^2}\gamma_{jd}^{-2} \\
&\propto (-1-1)\log \gamma_{jd}^2 - \frac{\lambda_{\text{feat}}}{2\phi_{jd}^2}\gamma_{jd}^2 - \frac{1}{2}\left[\lambda_{\text{feat}} + \mathbb{E}(\tau_d^{-2})\mathbb{E}(\sigma_d^{-2})\mathbb{E}(\beta_{jd}^2)\right]\gamma_{jd}^{-2}, \\
q_{\gamma_{jd}^2}(\gamma_{jd}^2) &\overset{D}{=} \mathcal{GIG}\left(-1, \lambda_{\text{feat}}/\phi_{jd}^2, \delta_{jd}\right), \\
&\quad \text{with } \delta_{jd} = \mathbb{E}(\tau_d^{-2})\mathbb{E}(\sigma_d^{-2})\left[\mathbb{E}(\beta_{jd})^2 + \mathbb{V}(\beta_{jd})\right] + \lambda_{\text{feat}}.
\end{aligned}$$

Similarly, we derive the variational posterior for τ_d^2 as:

$$\begin{aligned}
\log q_{\tau_d^2}(\tau_d^2) &\propto \mathbb{E}[\log \pi(\boldsymbol{\beta}_d|\boldsymbol{\gamma}_d^2, \sigma_d^2, \tau_d^2)] + \log \pi(\tau_d^2) \\
&\propto -\frac{p}{2}\log \tau_d^2 - \frac{1}{2}\sum_{j=1}^{p}\mathbb{E}\left(\frac{\beta_{jd}^2}{\sigma_d^2 \gamma_{jd}^2}\right)\tau_d^{-2} - \frac{3}{2}\log \tau_d^2 - \frac{\lambda_{\text{drug}}\{\tau_d^2 - \chi_d\}^2}{2\chi_d^2}\tau_d^{-2} \\
&\propto \left(-\frac{p+1}{2} - 1\right)\log \tau_d^2 - \frac{\lambda_{\text{drug}}}{2\chi_d^2}\tau_d^2 - \frac{1}{2}\left[\lambda_{\text{drug}} + \mathbb{E}(\sigma_d^{-2})\sum_{j=1}^{p}\mathbb{E}(\gamma_{jd}^{-2})\mathbb{E}(\beta_{jd}^2)\right]\tau_d^{-2}, \\
q_{\tau_d^2}(\tau_d^2) &\overset{D}{=} \mathcal{GIG}\left(-\frac{p+1}{2}, \lambda_{\text{drug}}/\chi_d^2, \eta_d\right), \\
&\quad \text{with } \eta_d = \mathbb{E}(\sigma_d^{-2})\sum_{j=1}^{p}\mathbb{E}(\gamma_{jd}^{-2})\left[\mathbb{E}(\beta_{jd})^2 + \mathbb{V}(\beta_{jd})\right] + \lambda_{\text{drug}}.
\end{aligned}$$

Lastly, we have the variational posterior for σ_d^2:

$$\begin{aligned}
\log q_{\sigma_d^2}(\sigma_d^2) &\propto \mathbb{E}[\log \mathcal{L}(\mathbf{y}_d|\boldsymbol{\beta}_d, \sigma_d^2)] + \mathbb{E}[\log \pi(\boldsymbol{\beta}_d|\boldsymbol{\gamma}_d^2, \sigma_d^2, \tau_d^2)] + \log \pi(\sigma_d^2) \\
&\propto -\frac{n}{2}\log \sigma_d^2 - \frac{1}{2}\sum_{i=1}^{n}\frac{\mathbb{E}[(y_{id} - \mathbf{x}_i^{\mathrm{T}}\boldsymbol{\beta}_d)^2]}{\sigma_d^2} - \frac{p}{2}\log \sigma_d^2 - \frac{1}{2}\sum_{j=1}^{p}\mathbb{E}\left(\frac{\beta_{jd}^2}{\gamma_{jd}^2 \tau_d^2}\right)\sigma_d^{-2} - \frac{3}{2}\log \sigma_d^2 \\
&= \left(-\frac{n+p+1}{2} - 1\right)\log \sigma_d^2 \\
&\quad - \frac{1}{2}\left\{\sum_{i=1}^{n}\mathbb{E}[(y_{id} - \mathbf{x}_i^{\mathrm{T}}\boldsymbol{\beta}_d)^2] + \mathbb{E}(\tau_d^{-2})\sum_{j=1}^{p}\mathbb{E}(\gamma_{jd}^{-2})\mathbb{E}(\beta_{jd}^2)\right\}\sigma_d^{-2}, \\
q_{\sigma_d^2}(\sigma_d^2) &\overset{D}{=} \Gamma^{-1}\left(\frac{n+p+1}{2}, \zeta_d\right), \\
&\quad \text{with } \zeta_d = \frac{1}{2}\Big\{\mathbf{y}_d^{\mathrm{T}}\mathbf{y}_d - 2\mathbf{y}_d^{\mathrm{T}}\mathbf{X}\mathbb{E}(\boldsymbol{\beta}_d) + \mathrm{tr}[\mathbf{X}^{\mathrm{T}}\mathbf{X}\mathbb{V}(\boldsymbol{\beta}_d)] + \mathbb{E}(\boldsymbol{\beta}_d^{\mathrm{T}})\mathbf{X}^{\mathrm{T}}\mathbf{X}\mathbb{E}(\boldsymbol{\beta}_d) \\
&\qquad + \mathbb{E}(\tau_d^{-2})\mathrm{tr}\{\mathrm{diag}[\mathbb{E}(\gamma_{jd}^{-2})]\mathbb{V}(\boldsymbol{\beta}_d)\} + \mathbb{E}(\tau_d^{-2})\mathbb{E}(\boldsymbol{\beta}_d^{\mathrm{T}})\mathrm{diag}[\mathbb{E}(\gamma_{jd}^{-2})]\mathbb{E}(\boldsymbol{\beta}_d)\Big\}.
\end{aligned}$$

If we fill in the expectations and variances, we arrive at the estimating equations in MD equations (3).

6 Evidence lower bound

The evidence lower bound that is maximised during the iterations is: $\text{ELBO}^{(l)} = \sum_{d=1}^{D} \text{ELBO}_d^{(l)}$, with:

$$
\begin{aligned}
&\text{ELBO}_d^{(l)} \\
&\quad = -\frac{n+p+1}{2}\log\pi + \frac{p+1-n}{2}\log 2 + \frac{n+1}{2} + p + \log\Gamma\left(\frac{n+p+1}{2}\right) \\
&\quad - \frac{n+p+1}{2}\log\zeta_d^{(l)} + \frac{1}{2}\log|\mathbf{\Sigma}_d^{(l)}| - \frac{n+p+1}{4\zeta_d^{(l)}}\Big[\mathbf{y}_d^{\mathrm{T}}\mathbf{y}_d - 2\mathbf{y}_d^{\mathrm{T}}\mathbf{X}\boldsymbol{\mu}_d^{(l)} + \mathrm{tr}(\mathbf{X}^{\mathrm{T}}\mathbf{X}\mathbf{\Sigma}_d^{(l)}) \\
&\quad + (\boldsymbol{\mu}_d^{(l)})^{\mathrm{T}}\mathbf{X}^{\mathrm{T}}\mathbf{X}\boldsymbol{\mu}_d^{(l)} + g_d^{(l)}\mathrm{tr}\left[\mathrm{diag}(b_{jd}^{(l)})\mathbf{\Sigma}_d^{(l)}\right] + g_d^{(l)}(\boldsymbol{\mu}_d^{(l)})^{\mathrm{T}}\mathrm{diag}(b_{jd}^{(l)})\boldsymbol{\mu}_d^{(l)}\Big] + p\log\lambda_{\text{feat}}^{(l)} \\
&\quad + \frac{p+3}{4}\log\lambda_{\text{drug}}^{(l)} + \frac{\eta_d^{(l)} - \lambda_{\text{drug}}^{(l)}}{2}g_d^{(l)} + \sum_{j=1}^{p}\frac{\delta_{jd}^{(l)} - \lambda_{\text{feat}}^{(l)}}{2}b_{jd}^{(l)} + \frac{\lambda_{\text{drug}}^{(l)}}{\chi_d^{(l)}} + \sum_{j=1}^{p}\frac{\lambda_{\text{feat}}^{(l)}}{\phi_{jd}^{(l)}} + \\
&\quad - \frac{p+1}{2}\log\chi_d^{(l)} - \sum_{j=1}^{p}\log\phi_{jd}^{(l)} - \frac{p+1}{4}\log\eta_d^{(l)} - \frac{1}{2}\sum_{j=1}^{p}\log\delta_{jd}^{(l)} \\
&\quad + \log K_{\frac{p+1}{2}}\left(\sqrt{\eta_d^{(l)}\lambda_{\text{drug}}^{(l)}/\chi_d^{(l)}}\right) + \sum_{j=1}^{p}\log K_1\left(\sqrt{\delta_{jd}^{(l)}\lambda_{\text{feat}}^{(l)}/\phi_{jd}^{(l)}}\right).
\end{aligned}
$$

7 Efficient computation

The empirical Bayes updates require the quantities e_{jd}, b_{jd}, g_d, and f_d, which in turn depend on the quantities $\mathrm{diag}(\mathbf{\Sigma}_d)$, $\boldsymbol{\mu}_d$, $\mathbf{y}_d^{\mathrm{T}}\mathbf{X}\boldsymbol{\mu}_d$, $\mathrm{tr}(\mathbf{X}^{\mathrm{T}}\mathbf{X}\mathbf{\Sigma}_d)$, $\boldsymbol{\mu}_d^{\mathrm{T}}\mathbf{X}^{\mathrm{T}}\mathbf{X}\boldsymbol{\mu}_d$, $g_d\mathrm{tr}[\mathrm{diag}(b_{jd})\mathbf{\Sigma}_d]$, and $g_d\boldsymbol{\mu}_d^{\mathrm{T}}\mathrm{diag}(b_{jd})\boldsymbol{\mu}_d$. Let $\mathbf{H}_d = g_d\mathrm{diag}[(b_{jd}^{(h)})^{-1}]$. Then, the first two quantities are efficiently calculated as:

$$
\begin{aligned}
\mathrm{diag}(\mathbf{\Sigma}_d^{(h+1)}) &= (a_d^{(h)})^{-1}\mathbf{H}_d^{(h)} - (a_d^{(h)})^{-1}\{[\mathbf{H}_d^{(h)}\mathbf{X}^{\mathrm{T}}(\mathbf{I}_n + \mathbf{X}\mathbf{H}_d^{(h)}\mathbf{X}^{\mathrm{T}})^{-1}] \circ (\mathbf{H}_d^{(h)}\mathbf{X}^{\mathrm{T}})\}\mathbf{1}_{n\times 1}, && \text{(1a)} \\
\boldsymbol{\mu}_d^{(h+1)}) &= \mathbf{H}_d^{(h)}\mathbf{X}^{\mathrm{T}}\mathbf{y}_d - \mathbf{H}_d^{(h)}\mathbf{X}^{\mathrm{T}}(\mathbf{I}_n + \mathbf{X}\mathbf{H}_d^{(h)}\mathbf{X}^{\mathrm{T}})^{-1}\mathbf{X}\mathbf{H}_d^{(h)}\mathbf{X}^{\mathrm{T}}\mathbf{y}_d. && \text{(1b)}
\end{aligned}
$$

The quantities $\mathbf{y}_d^{\mathrm{T}}\mathbf{X}\boldsymbol{\mu}_d$, $\boldsymbol{\mu}_d^{\mathrm{T}}\mathbf{X}^{\mathrm{T}}\mathbf{X}\boldsymbol{\mu}_d$, $g_d\mathrm{tr}[\mathrm{diag}(b_{jd})\mathbf{\Sigma}_d]$, and $g_d\boldsymbol{\mu}_d^{\mathrm{T}}\mathrm{diag}(b_{jd})\boldsymbol{\mu}_d$ are easily calculated from the first two. For the remaining one we have:

$$
\begin{aligned}
\mathrm{tr}(\mathbf{X}^{\mathrm{T}}\mathbf{X}\mathbf{\Sigma}_d^{(h+1)}) =& (a_d^{(h)})^{-1}\mathbf{1}_{1\times p}\mathbf{H}_d^{(h)}\mathbf{X}^{\mathrm{T}}\mathbf{1}_{n\times 1} \\
&- (a_d^{(h)})^{-1}\mathbf{1}_{1\times p}\{[\mathbf{H}_d^{(h)}\mathbf{X}^{\mathrm{T}}(\mathbf{I}_n + \mathbf{X}\mathbf{H}_d^{(h)}\mathbf{X}^{\mathrm{T}})^{-1}\mathbf{X}\mathbf{H}_d^{(h)}\mathbf{X}^{\mathrm{T}}] \circ \mathbf{X}^{\mathrm{T}}\}\mathbf{1}_{n\times 1}. \qquad (2)
\end{aligned}
$$

Both (1) and (2) are operations of complexity $\mathcal{O}(pn^2)$ instead of $\mathcal{O}(p^3)$ for naive calculation.

8 Ratios of modified Bessel functions

Ratios of modified Bessel functions of the second kind $K_{\alpha-1}(x)/K_\alpha(x)$ are prone to under- and overflow for large α. In our case, α increases linearly with p. Since p may be large, this may cause numerical issues in the calculation of various quantities. We alleviate the numerical issues through the following.

We let $n_1 = p/2$ and $n_2 = (p-1)/2$ and use the well-known recursive relation:

$$K_\alpha(x) = K_{\alpha-2}(x) + \frac{2(\alpha-1)}{2} K_{\alpha-1}(x),$$

to rewrite the ratio:

$$\frac{K_{\frac{p-1}{2}}(x)}{K_{\frac{p+1}{2}}(x)} = \begin{cases} \left(\dots\left(\left(1 + \frac{2\cdot 1-1}{x}\right)^{-1} + \frac{2\cdot 2-1}{x}\right)^{-1} + \dots + \frac{2\cdot n_1 - 1}{x}\right)^{-1}, & \text{for } p \text{ even,} \\ \left(\dots\left(\left(\frac{K_0(x)}{K_1(x)} + \frac{2}{x}\cdot 1\right)^{-1} + \frac{2}{x}\cdot 2\right)^{-1} + \dots + \frac{2}{x}\cdot n_2\right)^{-1}, & \text{for } p \text{ odd.} \end{cases}$$

The ratio $K_0(x)/K_1(x)$ is well-behaved, so that an arbitrary ratio $K_{\alpha-1}(x)/K_\alpha(x)$ may be calculated as a sequence of numerically stable scalar sums, products and inverses.

9 Empirical Bayes

For the feature scale parameter λ_{feat} updates to be non-negative, we have to show that:

$$\sum_{d=1}^{D}\sum_{j=1}^{p} b_{jd} + \boldsymbol{\alpha}_{\text{feat}}^{\text{T}}\mathbf{C}^{\text{T}}\text{diag}(e_{jd})\mathbf{C}\boldsymbol{\alpha}_{\text{feat}} - 2\boldsymbol{\alpha}_{\text{feat}}^{\text{T}}\mathbf{C}^{\text{T}}\mathbf{1}_{pD\times 1} = \sum_{d=1}^{D}\sum_{j=1}^{p}\left(b_{jd} + \phi_{jd}^{-2}e_{jd} - 2\phi_{jd}^{-1}\right) \geq 0, \tag{3}$$

which holds if all summands in the left-hand side of (3) are non-negative.

We note that through Jensen's inequality $e_{jd}^{-1} = \mathbb{E}(\gamma_{jd}^2)^{-1} \leq \mathbb{E}(\gamma_{jd}^{-2}) = b_{jd}$, we may lowerbound the summands in left-hand side of (3):

$$b_{jd} + \phi_{jd}^{-2}e_{jd} - 2\phi_{jd}^{-1} \geq e_{jd}^{-1} + \phi_{jd}^{-2}e_{jd} - 2\phi_{jd}^{-1}.$$

which we use to show that:

$$\begin{aligned} e_{jd}^{-1} + \phi_{jd}^{-2}e_{jd} - 2\phi_{jd}^{-1} &\geq 0 \\ 1 + \phi_{jd}^{-2}e_{jd}^2 - 2\phi_{jd}^{-1}e_{jd} &\geq 0 \\ \left(1 - \phi_{jd}^{-1}e_{jd}\right)^2 &\geq 0. \end{aligned}$$

Similar reasoning holds for the drug scale parameter λ_{drug}.

10 Gibbs sampler

MCMC samples from the posterior corresponding to model (2) may be generated for each equation independently. MCMC samples may be generated by iteratively sampling the following conditional

distributions:

$$\boldsymbol{\beta}_d|\boldsymbol{\gamma}_d^2, \sigma_d^2, \tau_d^2, \mathbf{y}_d \sim \mathcal{N}_p(\boldsymbol{\mu}_d, \boldsymbol{\Sigma}_d), \tag{4a}$$

$$\text{with } \boldsymbol{\Sigma}_d = \sigma_d^2[\mathbf{X}^{\mathrm{T}}\mathbf{X} + \tau_d^{-2}\text{diag}(\gamma_{jd}^{-2})]^{-1}, \tag{4b}$$

$$\text{and } \boldsymbol{\mu}_d = [\mathbf{X}^{\mathrm{T}}\mathbf{X} + \tau_d^{-2}\text{diag}(\gamma_{jd}^{-2})]^{-1}\mathbf{X}^{\mathrm{T}}\mathbf{y}_d, \tag{4c}$$

$$\gamma_{jd}^2|\beta_{jd}, \sigma_d^2, \tau_d^2, \mathbf{y} \sim \mathcal{GIG}\left(-1, \lambda_{\text{feat}}/\phi_{jd}^2, \delta_{jd}\right), \tag{4d}$$

$$\text{with } \delta_{jd} = \sigma_d^{-2}\tau_d^{-2}\beta_{jd}^2 + \lambda_{\text{feat}},$$

$$\tau_d^2|\boldsymbol{\beta}_d, \sigma_d^2, \boldsymbol{\gamma}_d^2, \mathbf{y} \sim \mathcal{GIG}\left(-\frac{p+1}{2}, \lambda_{\text{drug}}/\chi_d^2, \eta_d\right), \tag{4e}$$

$$\text{with } \eta_d = \sigma_d^{-2}\sum_{j=1}^{p}\gamma_{jd}^{-2}\beta_{jd}^2 + \lambda_{\text{drug}}, \tag{4f}$$

$$\sigma_d^2|\boldsymbol{\beta}_d, \boldsymbol{\gamma}_d^2, \tau_d^2, \mathbf{y} \sim \Gamma^{-1}\left(\frac{n+p+1}{2}, \zeta_d\right), \tag{4g}$$

$$\text{with } \zeta_d = \frac{1}{2}\left[\mathbf{y}_d^{\mathrm{T}}\mathbf{y}_d - 2\mathbf{y}_d^{\mathrm{T}}\mathbf{X}\boldsymbol{\beta}_d + \boldsymbol{\beta}_d\mathbf{X}^{\mathrm{T}}\mathbf{X}\boldsymbol{\beta}_d + \tau_d^{-2}\boldsymbol{\beta}_d^{\mathrm{T}}\text{diag}(\gamma_{jd}^{-2})\boldsymbol{\beta}_d)\right]. \tag{4h}$$

In high dimensional space, the $p \times p$ matrix inversions in (4b) and (4c) are significant computational bottlenecks. Bhattacharya et al. (2016) describe a method to sample from (4a) without explicit calculation of this inverse, thereby offering a siginifcant speed-up compared to naive sampling.

Sampling from (4) either requires estimating (as in MD Section 3.2) or specifying hyperparameters $\boldsymbol{\alpha}_{\text{feat}}$, $\boldsymbol{\alpha}_{\text{drug}}$, λ_{feat}, and λ_{drug}, or to endow them with and extra layer of hyperpriors. In general, specifying them requires rarely available, detailed subject knowledge, so we may resort to endowing them with hyperpriors (as in Upadhyay and Agrawal (1996)):

$$\begin{aligned}
\boldsymbol{\alpha}_{\text{feat},g}|\lambda_{\text{feat}} &\sim \mathcal{N}(0, \nu_{\text{feat}}^2/\lambda_{\text{feat}}),\\
\boldsymbol{\alpha}_{\text{drug},h}|\lambda_{\text{drug}} &\sim \mathcal{N}(0, \nu_{\text{drug}}^2/\lambda_{\text{drug}}),\\
\lambda_{\text{feat}} &\sim \Gamma(\kappa_{\text{feat}}, \xi_{\text{feat}}),\\
\lambda_{\text{drug}} &\sim \Gamma(\kappa_{\text{drug}}, \xi_{\text{drug}}),
\end{aligned}$$

with hyperparameters ν_{feat}^2, ν_{drug}^2, κ_{feat}, ξ_{feat}, κ_{drug}, and ξ_{drug}. Improper, flat priors occur if we let

$\nu_{\text{feat}}, \nu_{\text{drug}} \to \infty$ and $\kappa_{\text{feat}}, \kappa_{\text{drug}}, \xi_{\text{feat}}, \xi_{\text{drug}} \to 0$. The corresponding full conditionals are given by:

$$\begin{aligned}
\boldsymbol{\alpha}_{\text{feat}} | \lambda_{\text{feat}}, \boldsymbol{\Gamma} &\sim \mathcal{N}(\mathbf{m}_{\text{feat}}, \mathbf{S}_{\text{feat}}) \\
&\text{with } \mathbf{S}_{\text{feat}} = \lambda_{\text{feat}}^{-1} [\mathbf{C}^{\text{T}} \text{diag}(\gamma_{jd}^2) \mathbf{C} + \nu_{\text{feat}}^{-2} \mathbf{I}_G]^{-1}, \\
&\text{ and } \mathbf{m}_{\text{feat}} = [\mathbf{C}^{\text{T}} \text{diag}(\gamma_{jd}^2) \mathbf{C} + \nu_{\text{feat}}^{-2} \mathbf{I}_G]^{-1} \mathbf{C}^{\text{T}} \mathbf{1}_{pD \times 1}, \\
\boldsymbol{\alpha}_{\text{drug}} | \lambda_{\text{drug}}, \boldsymbol{\tau} &\sim \mathcal{N}(\mathbf{m}_{\text{drug}}, \mathbf{S}_{\text{drug}}), \\
&\text{with } \mathbf{S}_{\text{drug}} = \lambda_{\text{drug}}^{-1} [\mathbf{Z}^{\text{T}} \text{diag}(\tau_d^2) \mathbf{Z} + \nu_{\text{drug}}^{-2} \mathbf{I}_H]^{-1}, \\
&\text{ and } \mathbf{m}_{\text{drug}} = [\mathbf{Z}^{\text{T}} \text{diag}(\tau_d^2) \mathbf{Z} + \nu_{\text{drug}}^{-2} \mathbf{I}_H]^{-1} \mathbf{Z}^{\text{T}} \mathbf{1}_{D \times 1}, \\
\lambda_{\text{feat}} | \boldsymbol{\alpha}_{\text{feat}}, \boldsymbol{\Gamma} &\sim \Gamma\left(\frac{pD}{2} + \kappa_{\text{feat}}, \upsilon_{\text{feat}}\right), \\
&\text{with } \upsilon_{\text{feat}} = \frac{1}{2} \left[\mathbf{C}\boldsymbol{\alpha}_{\text{feat}} - \text{diag}(\gamma_{jd}^{-2}) \mathbf{1}_{pD \times 1}\right]^{\text{T}} \text{diag}(\gamma_{jd}^2) \left[\mathbf{C}\boldsymbol{\alpha}_{\text{feat}} - \text{diag}(\gamma_{jd}^{-2}) \mathbf{1}_{pD \times 1}\right] + \xi_{\text{feat}}, \\
\lambda_{\text{drug}} | \boldsymbol{\alpha}_{\text{drug}}, \boldsymbol{\tau} &\sim \Gamma\left(\frac{D}{2} + \kappa_{\text{drug}}, \upsilon_{\text{drug}}\right), \\
&\text{with } \upsilon_{\text{drug}} = \frac{1}{2} \left[\mathbf{Z}\boldsymbol{\alpha}_{\text{drug}} - \text{diag}(\tau_d^{-2}) \mathbf{1}_{D \times 1}\right]^{\text{T}} \text{diag}(\tau_d^2) \left[\mathbf{Z}\boldsymbol{\alpha}_{\text{drug}} - \text{diag}(\tau_d^{-2}) \mathbf{1}_{D \times 1}\right] + \xi_{\text{drug}},
\end{aligned}$$

which may be sampled together with (4).

11 Simulations

The lasso and ridge models in this section were fit separately per drug, using the `R` package `glmnet` (Friedman et al., 2010) with cross validated penalty parameters.

Tables 1-3 display averaged estimation mean squared error (EMSE), and prediction mean squared error (PMSE), calculated on independent test data, for simulation Scenarios 1-3. PMSE is calculated as in Section 5 of the MD, while EMSE is calculated as: EMSE $= D^{-1} p^{-1} \sum_{d=1}^{D} \sum_{j=1}^{p} (\beta_{jd} - \hat{\beta}_{jd})^2$, with $\hat{\beta}_{jd}$ the estimator for β_{jd}. In the NIG models, that provide the full posteriors, the posterior mean $\mathbb{E}(\beta_{jd} | \mathbf{y}_d)$ is used as point estimate. EMSE is further split into the contribution of the bottom 10% of the β_{jd} in terms of size (in absolute value) and the top 10% of the β_{jd} in size.

Focussing on estimation, we see that the NIG_{f}, NIG_{d}, and $\text{NIG}_{\text{f+d}}$ models (depending on the Scenario), that include the external covariates, outperform the other methods in terms of EMSE and PMSE. The lower EMSEs confirm that the NIG models that include the external data (NIG_{f}, NIG_{d}, and $\text{NIG}_{\text{f+d}}$) learn the underlying structure in the data better than the models that do not include the external data ($\text{NIG}_{\text{f}}^{-}$, $\text{NIG}_{\text{d}}^{-}$, and $\text{NIG}_{\text{f+d}}^{-}$). Furthermore, as expected, the sparser models lasso and NIG are better able to learn the β_{jd} with larger magnitude, as seen from the lower EMSE_{top}. On the other hand, ridge is better able to capture the smaller β_{jd}, as evident from the lower $\text{EMSE}_{\text{bottom}}$. Figure 1 shows that, as expected, the performance of the $\text{NIG}_{\text{f+d}}$ model deteriorates with increasing noise level in the external covariates and converges to the $\text{NIG}_{\text{f+d}}^{-}$ model performance.

Table 1: Mean EMSE (separated for bottom and top 10% of $|\hat{\beta}_{jd}|$) and PMSE for simulation Scenario 1, estimated on test data (lowest in bold).

	$\text{EMSE}_{\text{total}}$	EMSE_{top}	$\text{EMSE}_{\text{bottom}}$	PMSE
$\text{NIG}_{\text{f}}^{-}$	0.349 (0.024)	2.135 (0.163)	**0.001 (0)**	0.033 (0.001)
NIG_{f}	**0.348 (0.024)**	**2.133 (0.163)**	0.001 (0)	**0.033 (0.001)**
ridge	0.371 (0.025)	2.284 (0.168)	0.002 (0)	0.045 (0.001)
lasso	0.349 (0.024)	2.134 (0.163)	0.001 (0)	0.033 (0.001)

Table 2: Mean EMSE (separated for bottom and top 10% of $|\hat{\beta}_{jd}|$) and PMSE for simulation Scenario 2, estimated on test data (lowest in bold).

	$\text{EMSE}_{\text{total}}$	EMSE_{top}	$\text{EMSE}_{\text{bottom}}$	PMSE
$\text{NIG}_{\text{d}}^{-}$	0.359 (0.037)	1.552 (0.161)	**0.003 (0)**	0.053 (0.001)
NIG_{d}	**0.359 (0.037)**	1.552 (0.161)	0.003 (0)	**0.053 (0.001)**
ridge	0.376 (0.038)	1.626 (0.164)	0.003 (0)	0.062 (0.002)
lasso	0.359 (0.037)	**1.548 (0.161)**	0.003 (0)	0.053 (0.001)

Table 3: Mean EMSE (separated for bottom and top 10% of $|\hat{\beta}_{jd}|$) and PMSE for simulation Scenario 3, estimated on test data (lowest in bold).

	$\text{EMSE}_{\text{total}}$	EMSE_{top}	$\text{EMSE}_{\text{bottom}}$	PMSE
$\text{NIG}_{\text{f+d}}^{-}$	0.157 (0.025)	0.955 (0.159)	0.001 (0)	0.127 (0.004)
$\text{NIG}_{\text{f+d}}$	**0.157 (0.025)**	0.953 (0.159)	0.001 (0)	**0.126 (0.004)**
ridge	0.167 (0.026)	1.025 (0.163)	0.001 (0)	0.134 (0.004)
lasso	0.157 (0.025)	**0.951 (0.159)**	**0.001 (0)**	0.128 (0.004)

Figure 2 shows instances of both MCMC and VB β_{jd} posteriors from simulation Scenario 3 for the $\text{NIG}_{\text{f+d}}$ model. The chosen β_{jd} represent every combination of four drugs and four feature groups (for a total of 16 β_{jd}). The Figure shows that the MCMC and variational posteriors are in agreement. This leads us to conclude that the variational approximation to the posterior is quite accurate in this Scenario. The simulation Scenario was setup to mimic the real GDSC data, so that we expect an accurate variational posterior in the real GDSC data as well.

For completeness, Figure 3 displays samples from the full Bayesian NIG posterior, as introduced in Section 10. Unfortunately, we were not able to generate enough samples for a reliable posterior using the Gibbs procedure as described in the Section. We generated the samples from the full Bayesian posterior using stan through the R package `rstan` (Guo et al., 2018) (see `https://github.com/magnusmunch/NIG` for the implemented stan code). The Figure shows that the posteriors for $\boldsymbol{\phi}$ and $\boldsymbol{\chi}$ have unexpected shapes and seem to have explored only a small part of the posterior space. This indication that posterior inference is not reliable is confirmed by the average and minimum effective sample sizes of 66 and 2over all parameters, respectively. For reliable inference, Kruschke (2015) recommends an effective sample size of at least 10 000. Inspection of the computation times and a quick calculation learns that we have to sample from the posterior for at least 37 hours to obtain an average effective sample size of 10 000. Moreover, to achieve a

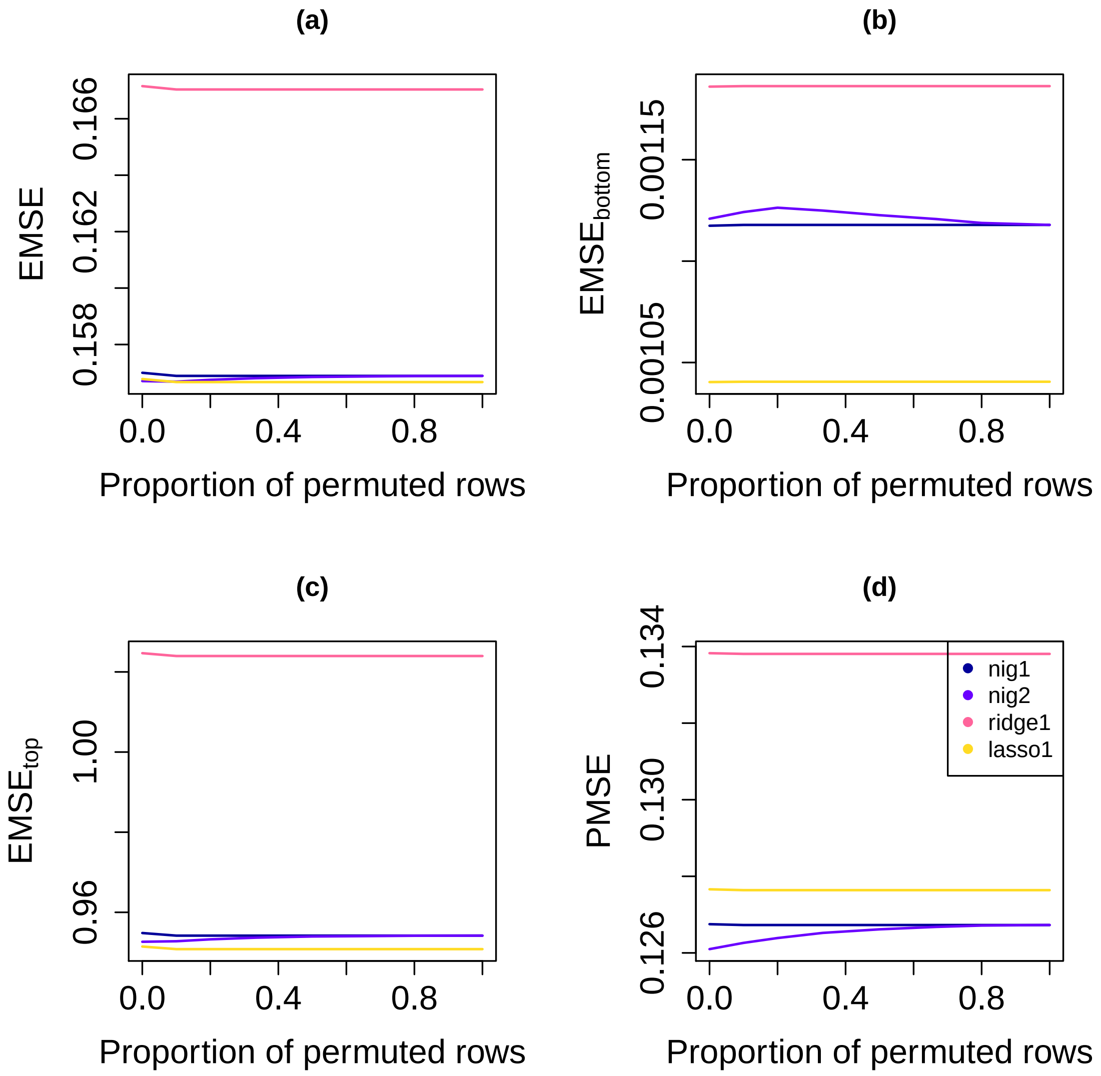

Figure 1: (a) Mean EMSE, separated for (b) bottom and (c) top 10% of $|\hat{\beta}_{jd}|$, and (d) PMSE for simulation Scenario 4 versus the proportion of permuted rows in the external data, estimated on test data.

minimum effective sample size of 10 000 for all parameters, the sampler has to be run for about 7 weeks.

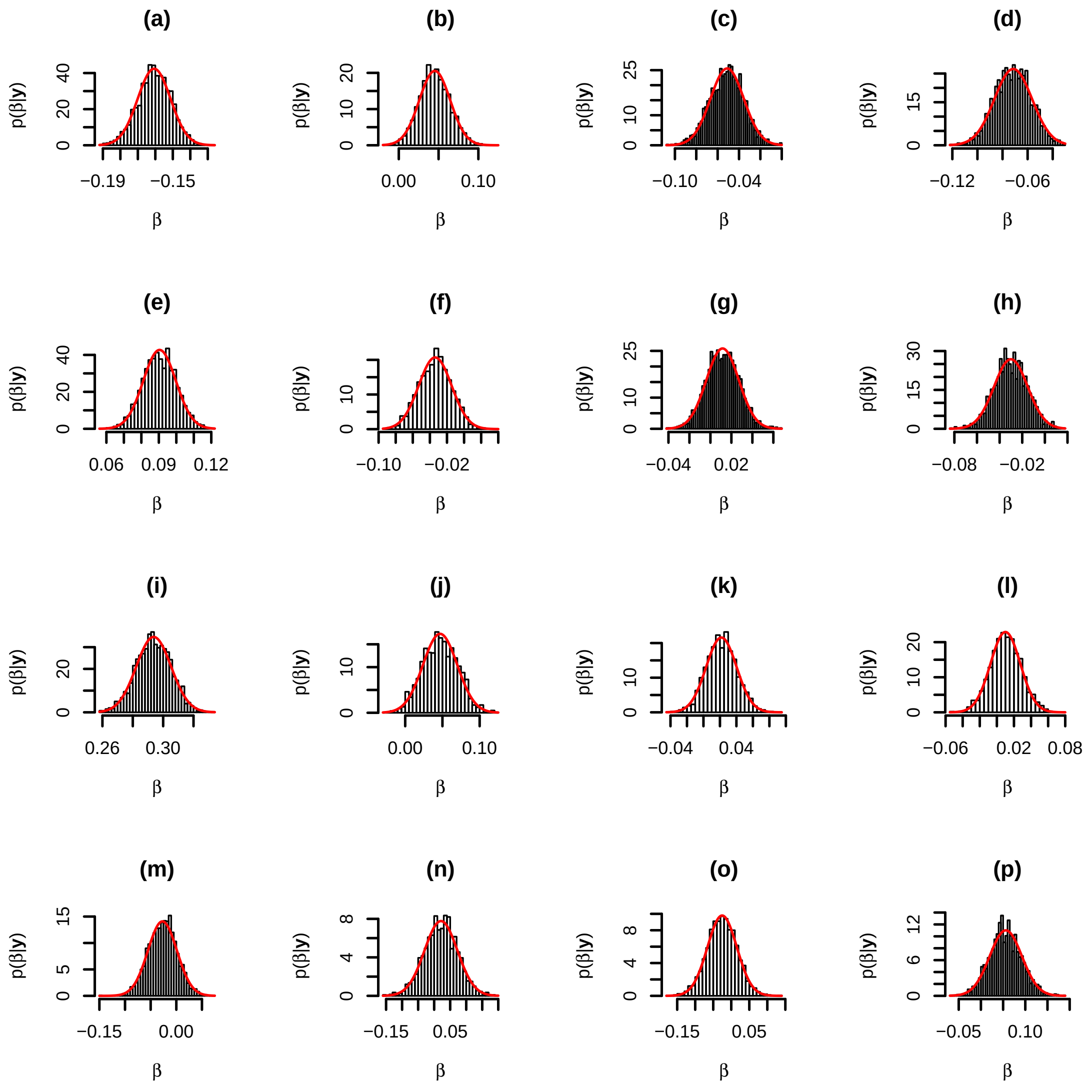

Figure 2: Simulation results for Scenario 3: Samples from $\mathrm{NIG}_{\mathrm{f+d}}$ posterior with variational posterior superimposed (red line) for estimated hyperparameters (top row) $\hat{\chi}_1$, (second row) $\hat{\chi}_2$, (third row) $\hat{\chi}_3$, (bottom row) $\hat{\chi}_4$, (left column) $\hat{\phi}_1$, (second column) $\hat{\phi}_2$, (third column) $\hat{\phi}_3$, and (last column) $\hat{\phi}_4$.

12 GDSC data

the CPO for cell line i and drug d is

$$\mathrm{CPO}_{id} = p(y_{id}|\boldsymbol{y}_{-id}) = \left[\int_{\boldsymbol{\beta}_d} \int_{\sigma_d^2} \mathcal{L}(\mathbf{y}_{id}|\boldsymbol{\beta}_d, \sigma_d^2)^{-1} q(\boldsymbol{\beta}_d|\boldsymbol{y}_d) q(\sigma_d^2|\boldsymbol{y}_d) d\boldsymbol{\beta}_d \sigma_d^2 \right]^{-1} \quad (5)$$

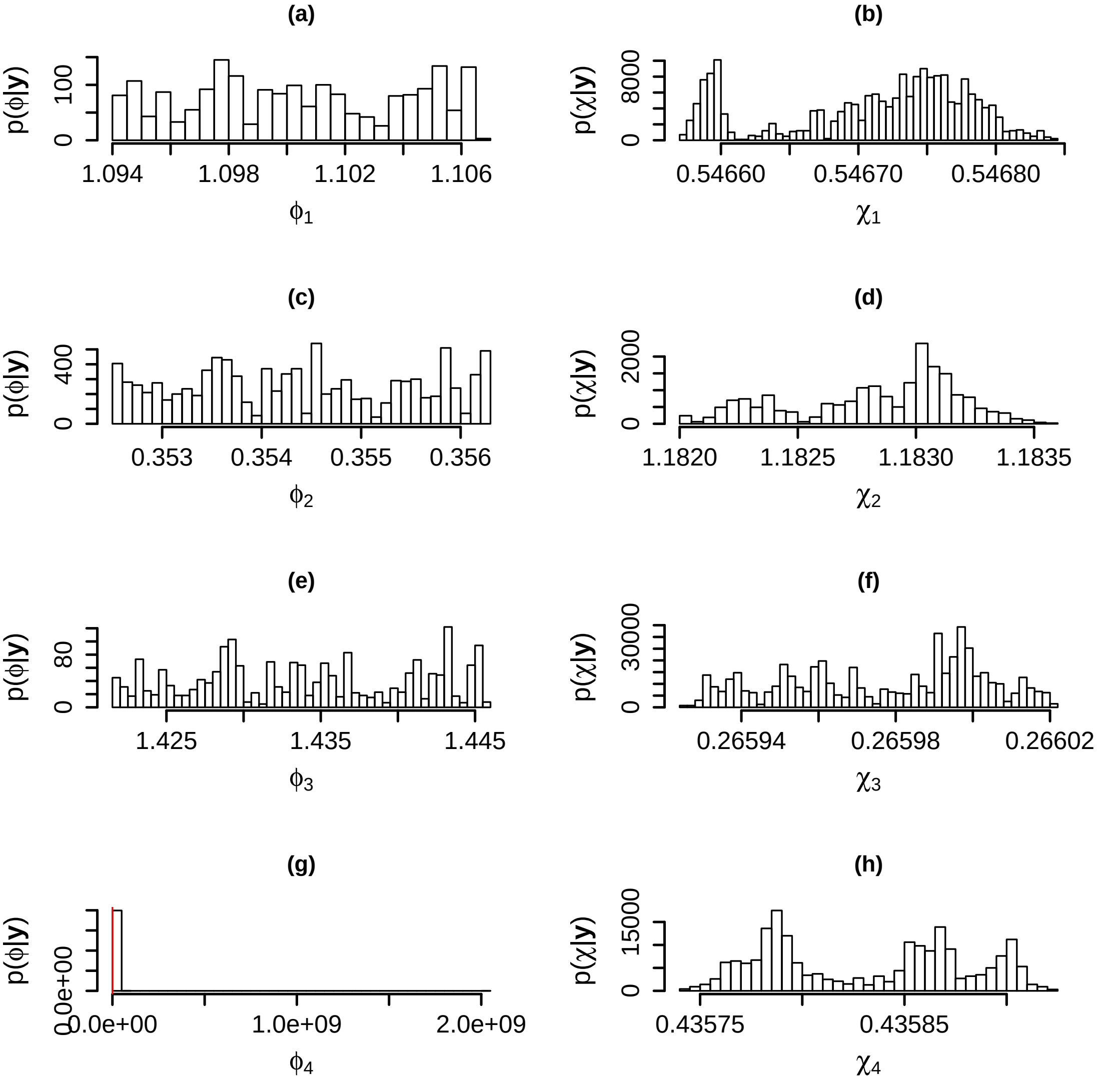

Figure 3: Simulation results for Scenario 3: Samples from full Bayesian NIG posteriors (see Section 10) with true value superimposed (red line) for (top row) group 1, (second row) group 2, (third row) group 3, and (bottom row) group 4 for both features and drugs, and (left column) $\boldsymbol{\phi}$ and (second column) $\boldsymbol{\chi}$. Note that the red line is not present in most of the Figures, indicating that the posterior is far of from the true value

and is the Bayesian version of the leave-one-out cross-validated likelihood. Congdon (2005) suggests as outlier cut-off 0.01. Figure 4 displays these CPOs (calculated by numerical integration of (5)) for the four Analyses. For $y_{id}|y_{-id}$ following a Gaussian distribution with standard deviation 0.83 (the average posterior mean of σ_d^2 from our analyses) this implies an expected proportion of outliers of about 0.003. In our analyses we find 0.006, 0.006, 0.006, and 0.006, for the four analyses respectively. So about double the amount of expected outliers under a Gaussian posterior

predictive distribution. However, in reality, due to posterior uncertainty of $\boldsymbol{\beta}_d$ and σ_d^2, the predictive distribution is more heavy-tailed than the Gaussian and the corresponding expected proportion of outliers higher than 0.003. Unfortunately, calculation of this true expected proportion is infeasible due to intractibility of the posterior predictive distribution.

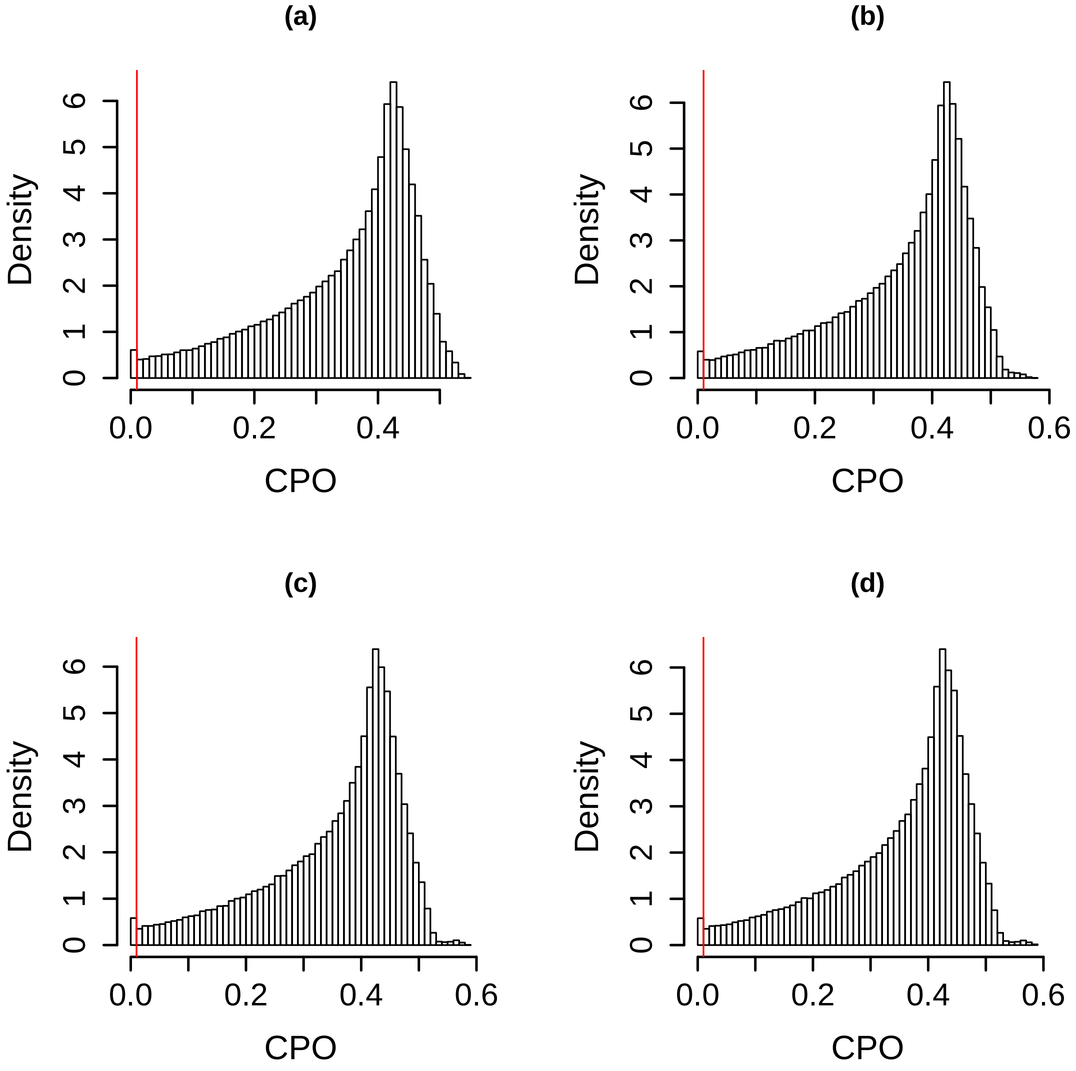

Figure 4: distribution of the CPO values for Analysis (a) 1, (b) 2, (c) 3, and (d) 4 in Section 5 of the MD, with outlier cutoff value 0.01 (red line).

References

Bhattacharya, A. et al. (2016). Fast sampling with Gaussian scale mixture priors in high-dimensional regression. *Biometrika*, **103**, 985–991.

Congdon, P.D. (2005). *Bayesian models for categorical data*. Wiley series in probability and mathematical statistics 810651580. John Wiley, Chichester [etc.].

Friedman, J. et al. (2010). Regularization paths for generalized linear models via coordinate descent. *Journal of Statistical Software*, **33**, 1–22.

Guo, J. et al. (2018). rstan: R Interface to Stan.
URL `https://CRAN.R-project.org/package=rstan`

Jeffreys, H. (1946). An Invariant Form for the Prior Probability in Estimation Problems. *Proceedings of the Royal Society of London A: Mathematical, Physical and Engineering Sciences*, **186**, 453–461.

Kruschke, J.K. (2015). *Doing Bayesian data analysis*. Elsevier, second edition edition.

Upadhyay, S.K. and Agrawal, R. (1996). Bayesian Analysis of Inverse Gaussian Non-Linear Regression by Simulation. *Sankhyā: The Indian Journal of Statistics, Series B*, **58**, 363–378.

Session info

```
devtools::session_info()
```

```
## - Session info ---------------------------------------------------------------
##  setting  value
##  version  R version 3.6.3 (2020-02-29)
##  os       macOS Catalina 10.15.3
##  system   x86_64, darwin15.6.0
##  ui       X11
##  language (EN)
##  collate  en_US.UTF-8
##  ctype    en_US.UTF-8
##  tz       Europe/Brussels
##  date     2020-04-30
##
## - Packages -------------------------------------------------------------------
##  package     * version date       lib source
##  assertthat    0.2.1   2019-03-21 [1] CRAN (R 3.6.0)
##  backports     1.1.6   2020-04-05 [1] CRAN (R 3.6.2)
##  callr         3.4.3   2020-03-28 [1] CRAN (R 3.6.2)
##  cli           2.0.2   2020-02-28 [1] CRAN (R 3.6.0)
##  colorspace    1.4-1   2019-03-18 [1] CRAN (R 3.6.0)
##  crayon        1.3.4   2017-09-16 [1] CRAN (R 3.6.0)
##  desc          1.2.0   2018-05-01 [1] CRAN (R 3.6.0)
##  devtools      2.3.0   2020-04-10 [1] CRAN (R 3.6.3)
##  digest        0.6.25  2020-02-23 [1] CRAN (R 3.6.0)
##  ellipsis      0.3.0   2019-09-20 [1] CRAN (R 3.6.0)
##  evaluate      0.14    2019-05-28 [1] CRAN (R 3.6.0)
##  fansi         0.4.1   2020-01-08 [1] CRAN (R 3.6.0)
##  formatR       1.7     2019-06-11 [1] CRAN (R 3.6.0)
##  fs            1.4.1   2020-04-04 [1] CRAN (R 3.6.2)
##  glue          1.4.0   2020-04-03 [1] CRAN (R 3.6.2)
```

```
##  hms           0.5.3   2020-01-08 [1] CRAN (R 3.6.0)
##  htmltools     0.4.0   2019-10-04 [1] CRAN (R 3.6.0)
##  httr          1.4.1   2019-08-05 [1] CRAN (R 3.6.0)
##  kableExtra    1.1.0   2019-03-16 [1] CRAN (R 3.6.0)
##  knitr       * 1.28    2020-02-06 [1] CRAN (R 3.6.0)
##  lattice       0.20-38 2018-11-04 [1] CRAN (R 3.6.3)
##  lifecycle     0.2.0   2020-03-06 [1] CRAN (R 3.6.0)
##  magrittr      1.5     2014-11-22 [1] CRAN (R 3.6.0)
##  memoise       1.1.0   2017-04-21 [1] CRAN (R 3.6.0)
##  munsell       0.5.0   2018-06-12 [1] CRAN (R 3.6.0)
##  pillar        1.4.3   2019-12-20 [1] CRAN (R 3.6.0)
##  pkgbuild      1.0.6   2019-10-09 [1] CRAN (R 3.6.0)
##  pkgconfig     2.0.3   2019-09-22 [1] CRAN (R 3.6.0)
##  pkgload       1.0.2   2018-10-29 [1] CRAN (R 3.6.0)
##  prettyunits   1.1.1   2020-01-24 [1] CRAN (R 3.6.0)
##  processx      3.4.2   2020-02-09 [1] CRAN (R 3.6.0)
##  ps            1.3.2   2020-02-13 [1] CRAN (R 3.6.0)
##  R6            2.4.1   2019-11-12 [1] CRAN (R 3.6.0)
##  Rcpp          1.0.4.6 2020-04-09 [1] CRAN (R 3.6.3)
##  readr         1.3.1   2018-12-21 [1] CRAN (R 3.6.0)
##  remotes       2.1.1   2020-02-15 [1] CRAN (R 3.6.0)
##  rlang         0.4.5   2020-03-01 [1] CRAN (R 3.6.0)
##  rmarkdown     2.1     2020-01-20 [1] CRAN (R 3.6.0)
##  rprojroot     1.3-2   2018-01-03 [1] CRAN (R 3.6.0)
##  rstudioapi    0.11    2020-02-07 [1] CRAN (R 3.6.0)
##  rvest         0.3.5   2019-11-08 [1] CRAN (R 3.6.0)
##  scales        1.1.0   2019-11-18 [1] CRAN (R 3.6.0)
##  sessioninfo   1.1.1   2018-11-05 [1] CRAN (R 3.6.0)
##  sp          * 1.4-1   2020-02-28 [1] CRAN (R 3.6.0)
##  stringi       1.4.6   2020-02-17 [1] CRAN (R 3.6.0)
##  stringr       1.4.0   2019-02-10 [1] CRAN (R 3.6.0)
##  testthat      2.3.2   2020-03-02 [1] CRAN (R 3.6.0)
##  tibble        3.0.0   2020-03-30 [1] CRAN (R 3.6.2)
##  usethis       1.6.0   2020-04-09 [1] CRAN (R 3.6.3)
##  vctrs         0.2.4   2020-03-10 [1] CRAN (R 3.6.0)
##  viridisLite   0.3.0   2018-02-01 [1] CRAN (R 3.6.0)
##  webshot       0.5.2   2019-11-22 [1] CRAN (R 3.6.0)
##  withr         2.1.2   2018-03-15 [1] CRAN (R 3.6.0)
##  xfun          0.13    2020-04-13 [1] CRAN (R 3.6.2)
##  xml2          1.3.1   2020-04-09 [1] CRAN (R 3.6.2)
##
## [1] /Library/Frameworks/R.framework/Versions/3.6/Resources/library
```
