# Supplementary figures and images for: Drug sensitivity prediction with normal inverse Gaussian shrinkage informed by external data
Source: Biom J. 2020 Jul 23;63(2):289–304. doi: 10.1002/bimj.201900371 (PMC7891636; doi:10.1002/bimj.201900371)

**(a)**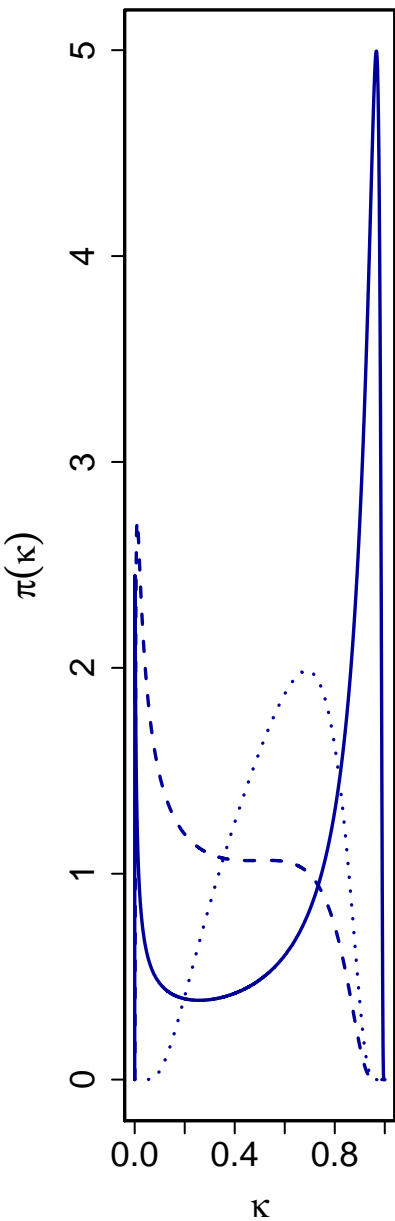**(b)**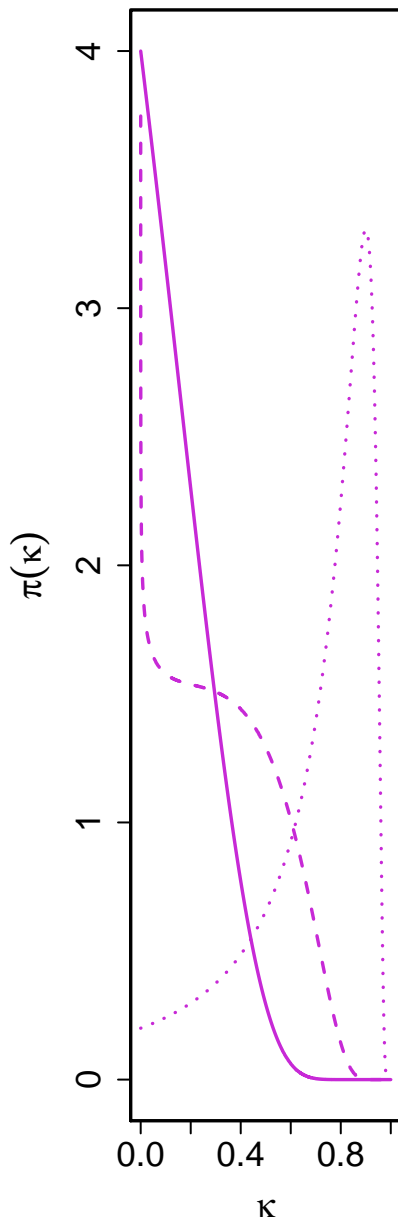**(c)**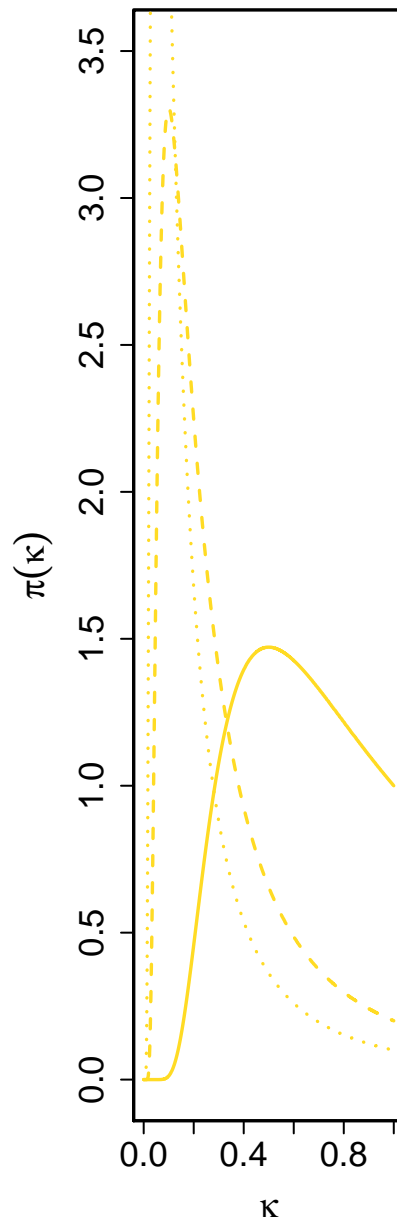

Supplement: Supplementary file 2 — Supporting Information [file BIMJ-63-289-s001.zip › Code_and_Data/Code_and_Data/figures/main_figure1.pdf]

(a)

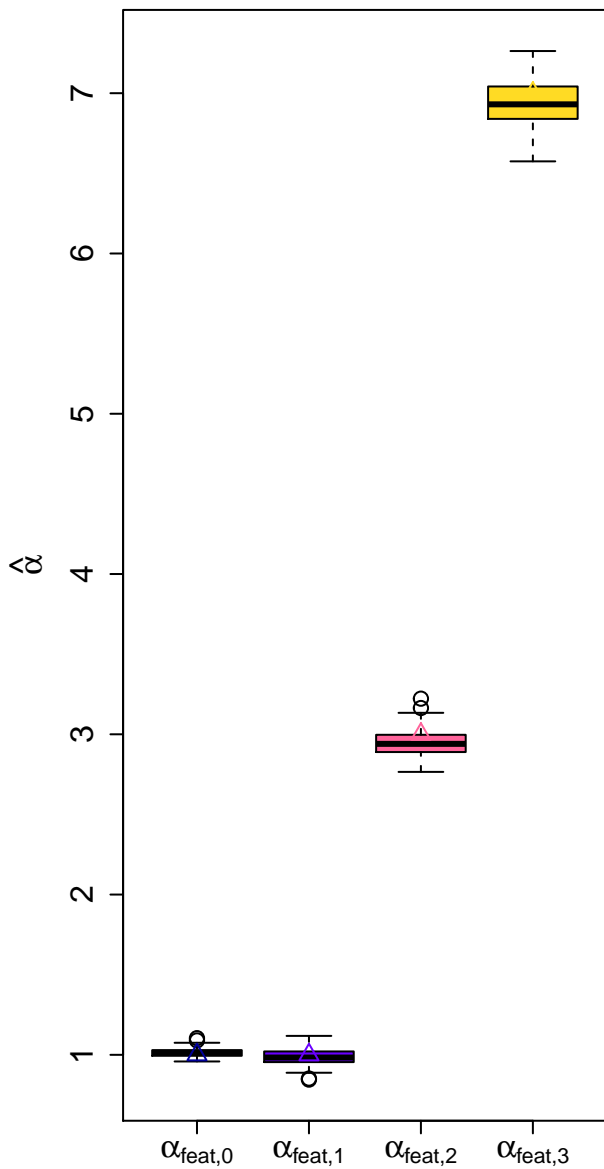

(b)

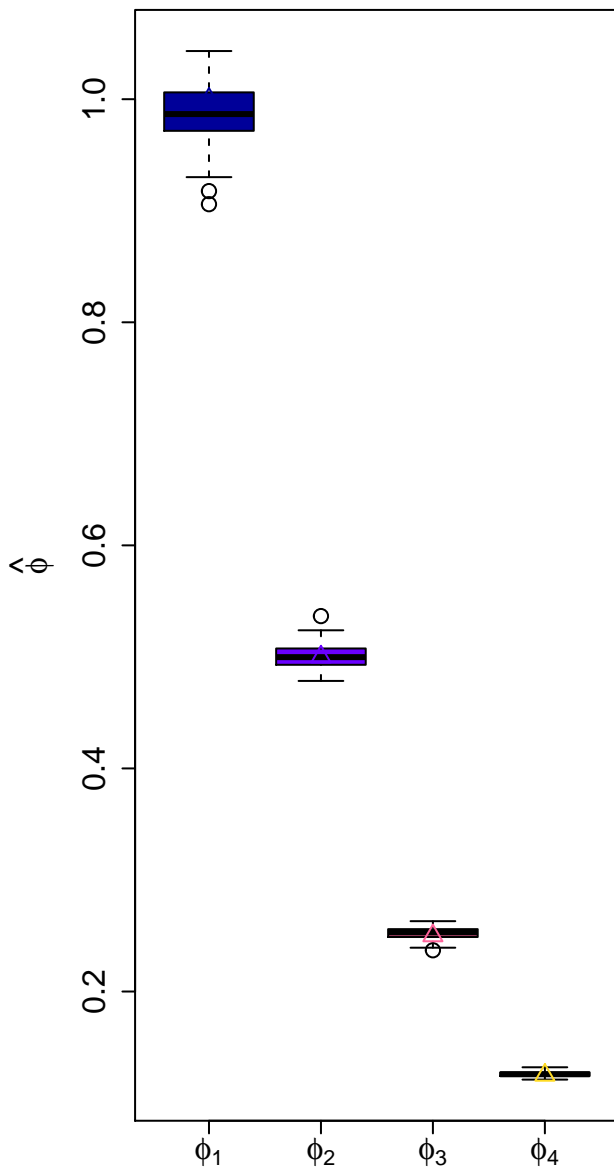

Supplement: Supplementary file 2 — Supporting Information [file BIMJ-63-289-s001.zip › Code_and_Data/Code_and_Data/figures/main_figure3.pdf]

(a)

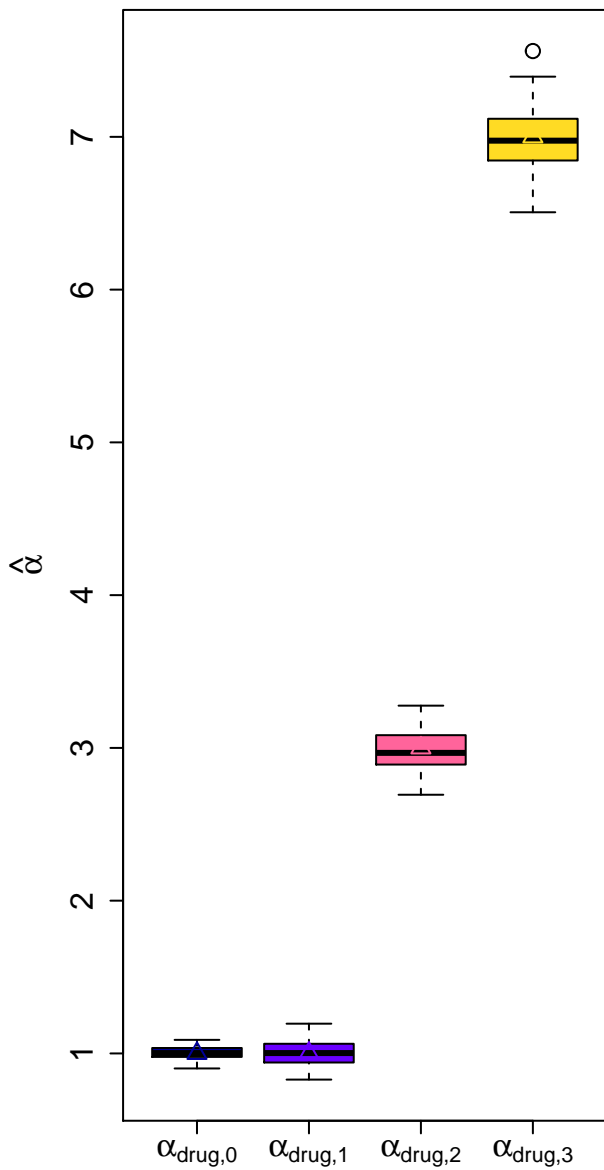

(b)

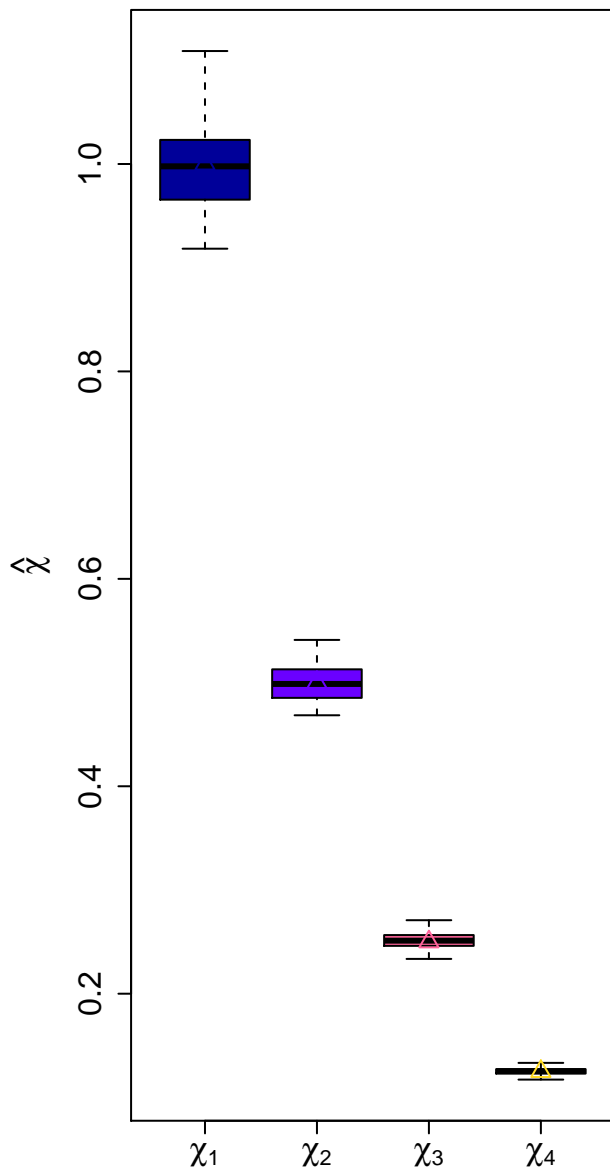

Supplement: Supplementary file 2 — Supporting Information [file BIMJ-63-289-s001.zip › Code_and_Data/Code_and_Data/figures/main_figure4.pdf]

(a)

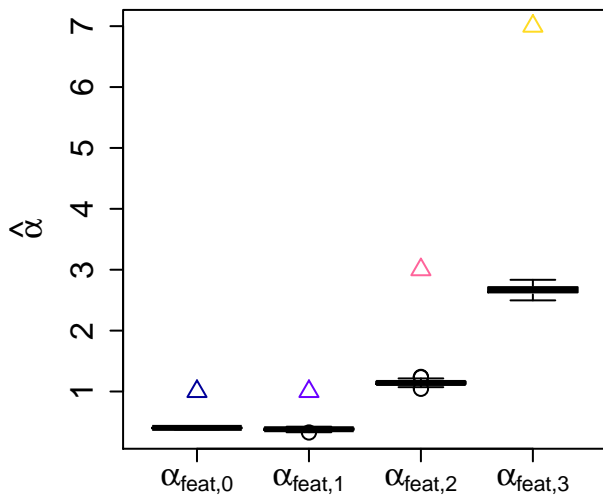

(b)

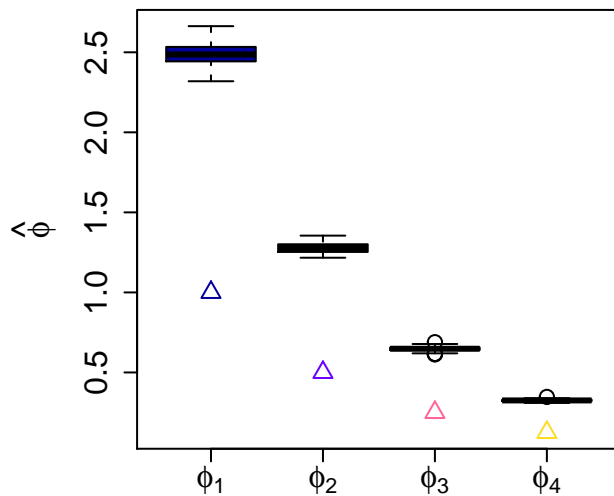

(c)

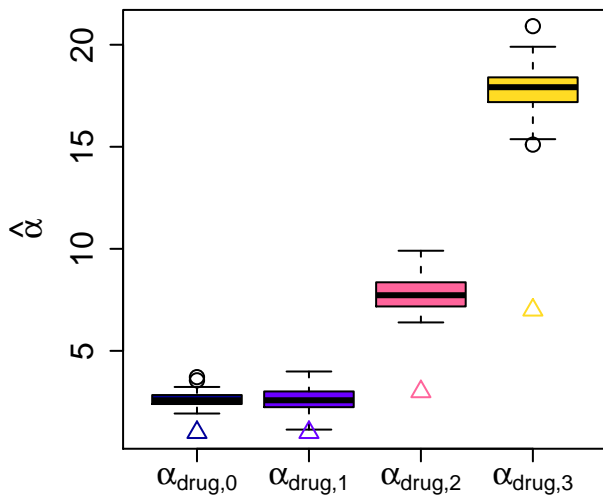

(d)

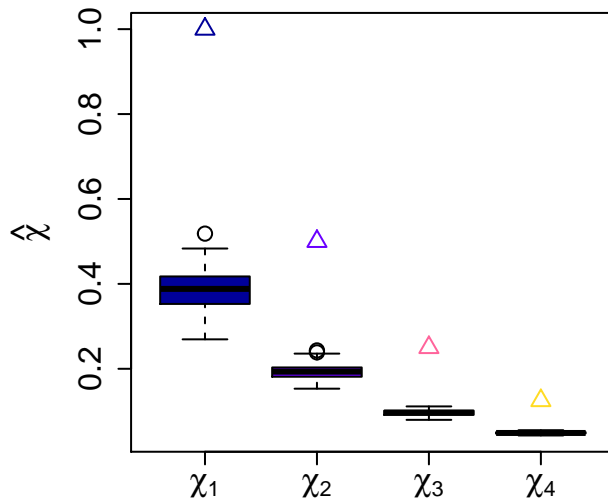

Supplement: Supplementary file 2 — Supporting Information [file BIMJ-63-289-s001.zip › Code_and_Data/Code_and_Data/figures/main_figure5.pdf]

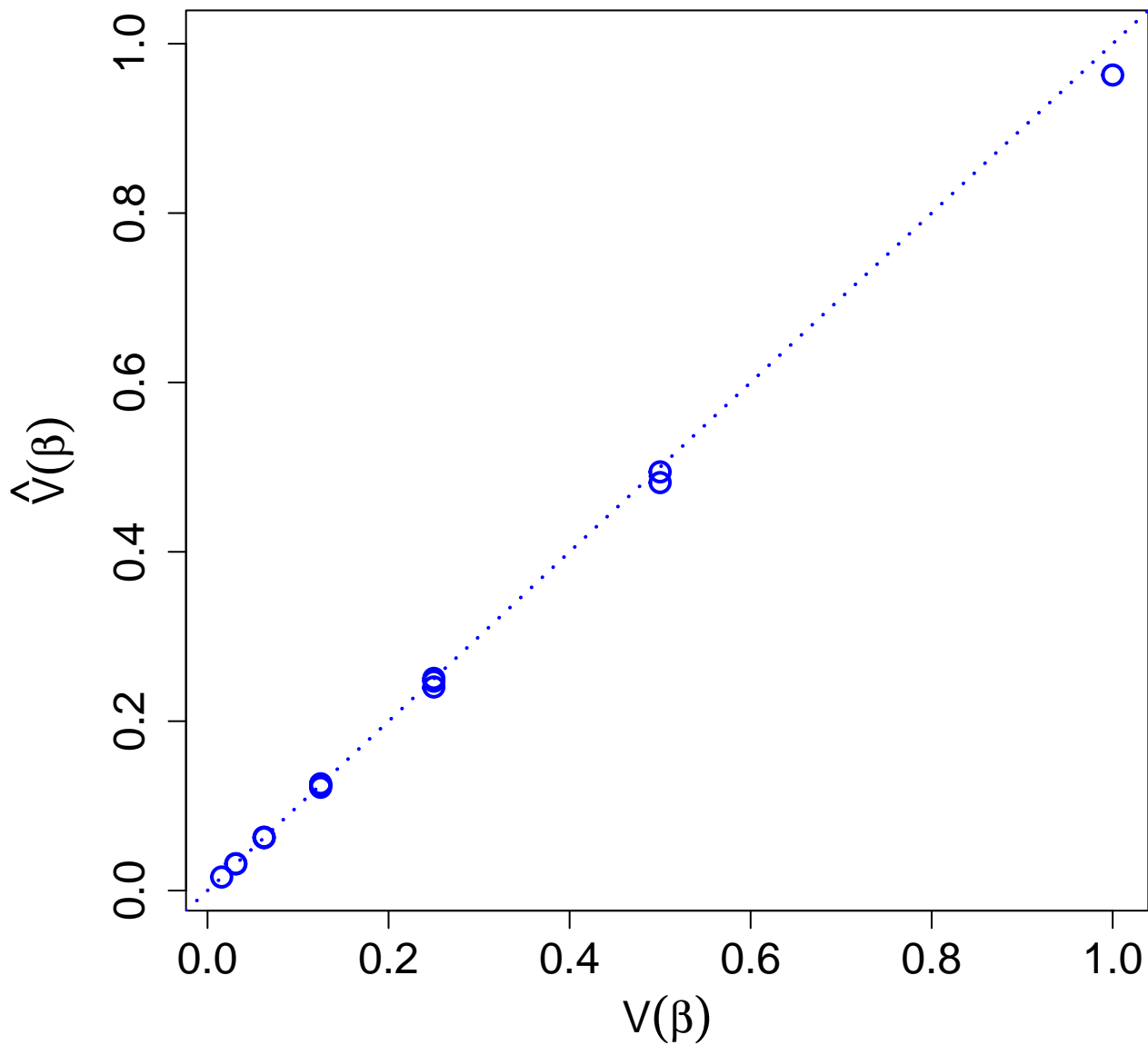

Supplement: Supplementary file 2 — Supporting Information [file BIMJ-63-289-s001.zip › Code_and_Data/Code_and_Data/figures/main_figure6.pdf]

(a)

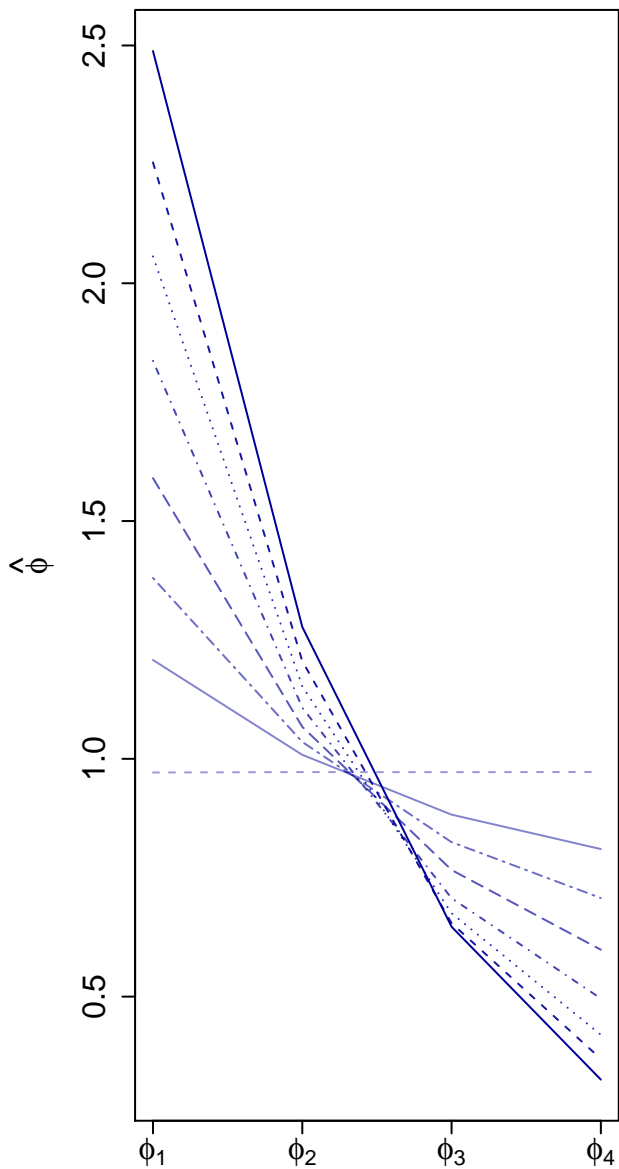

(b)

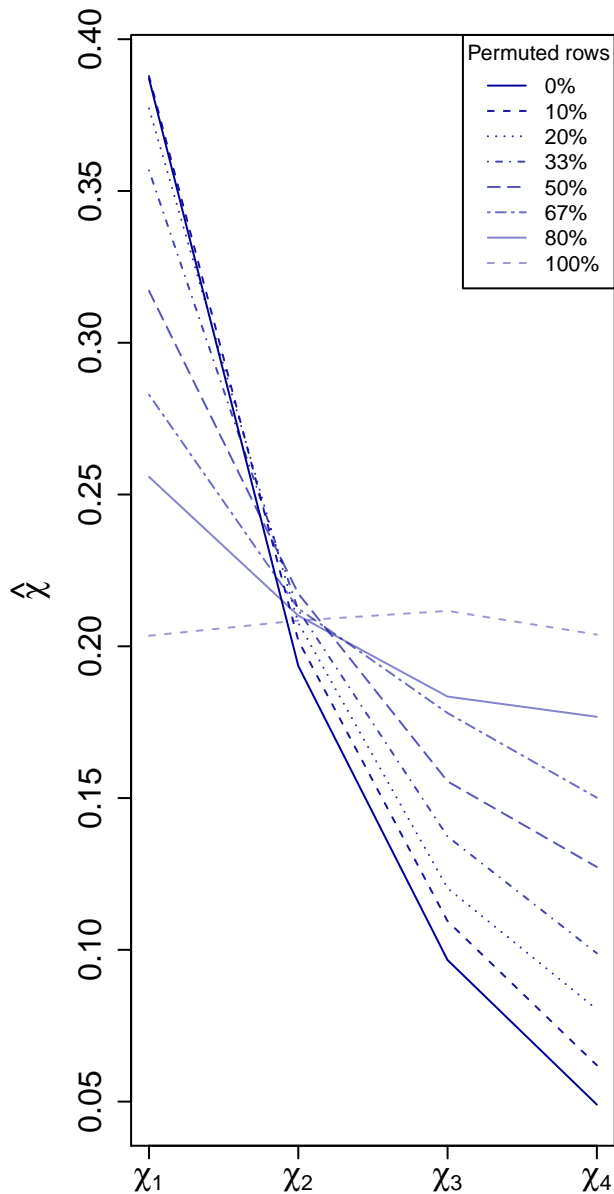

Supplement: Supplementary file 2 — Supporting Information [file BIMJ-63-289-s001.zip › Code_and_Data/Code_and_Data/figures/main_figure7.pdf]

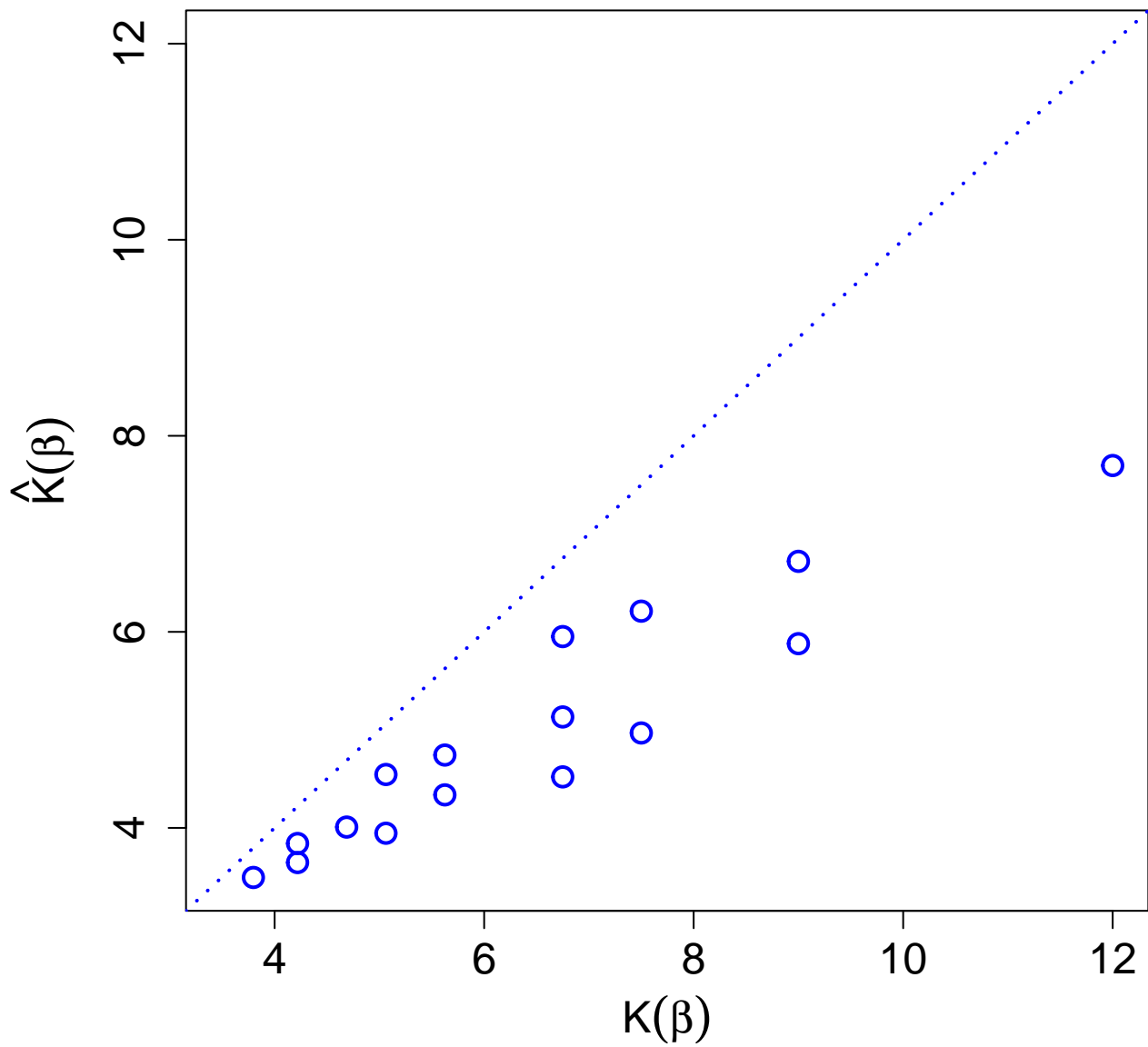

Supplement: Supplementary file 2 — Supporting Information [file BIMJ-63-289-s001.zip › Code_and_Data/Code_and_Data/figures/main_figure8.pdf]

(a)

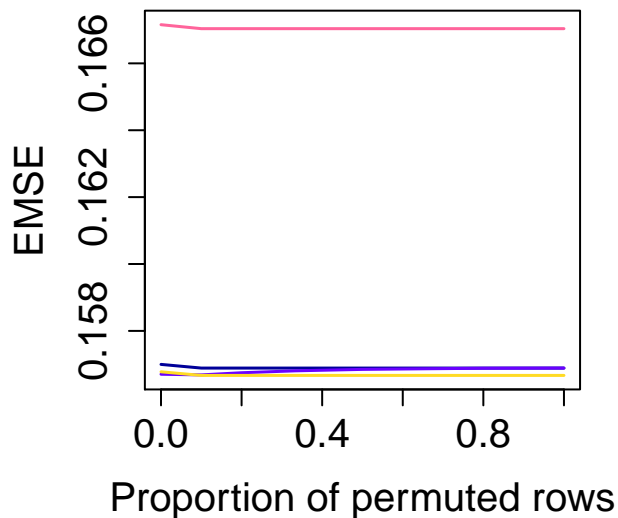

(b)

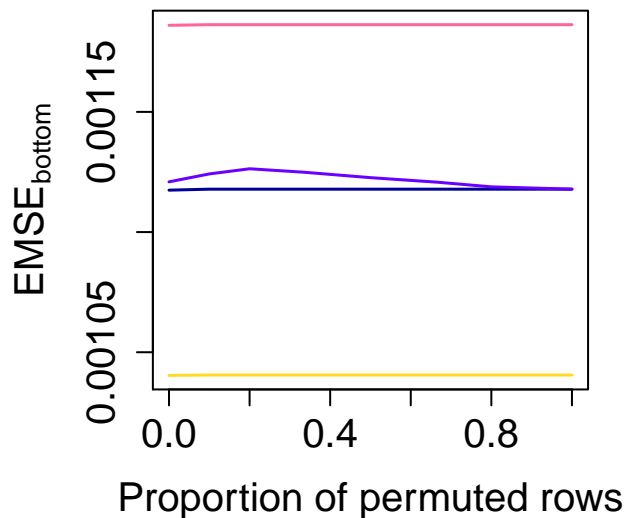

(c)

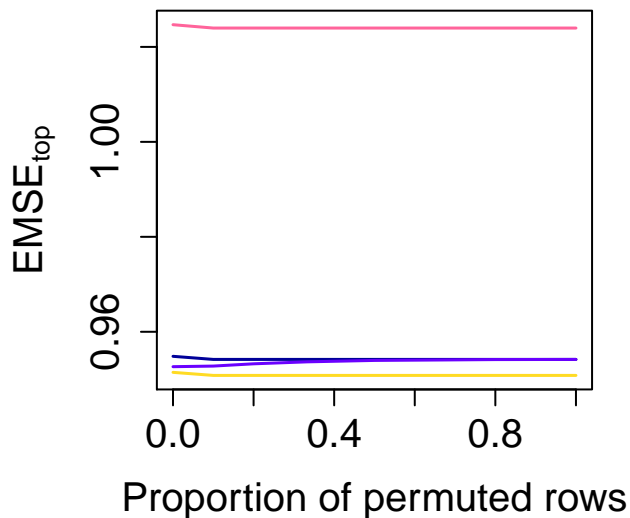

(d)

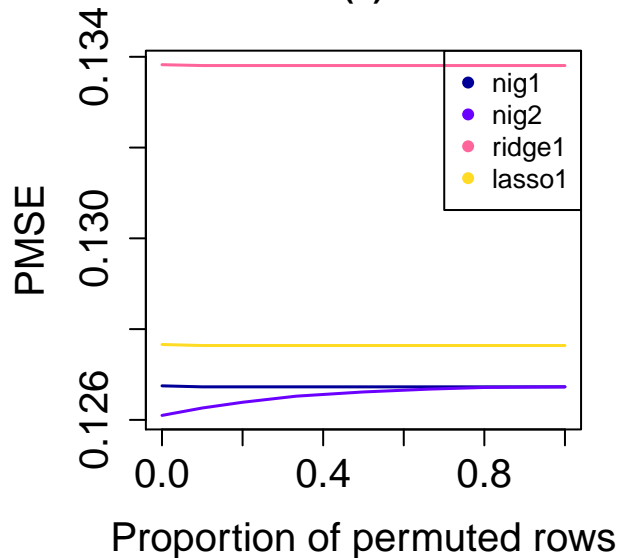

Supplement: Supplementary file 2 — Supporting Information [file BIMJ-63-289-s001.zip › Code_and_Data/Code_and_Data/figures/supp_figure1.pdf]

**(a)**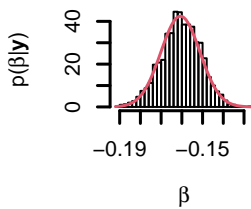**(b)**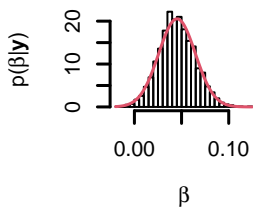**(c)**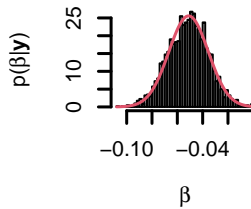**(d)**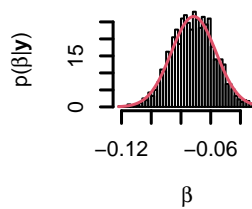**(e)**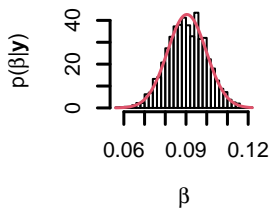**(f)**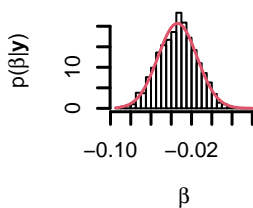**(g)**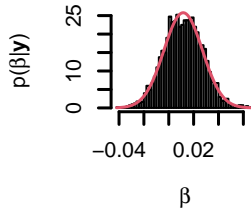**(h)**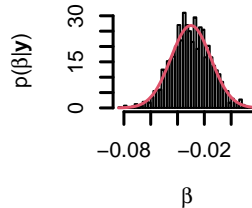**(i)**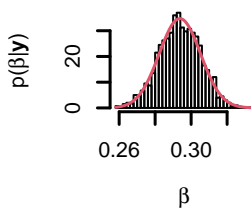**(j)**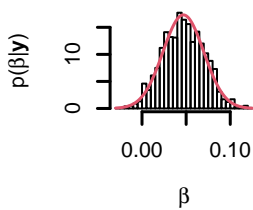**(k)**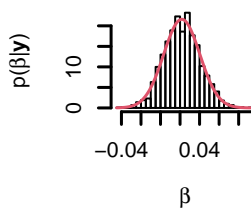**(l)**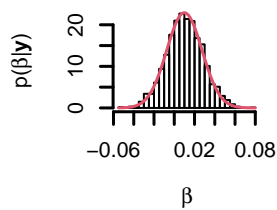**(m)**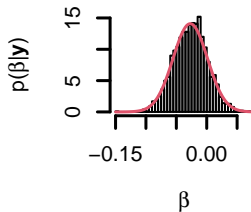**(n)**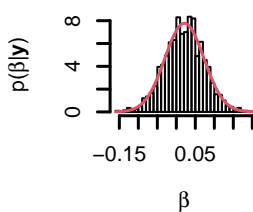**(o)**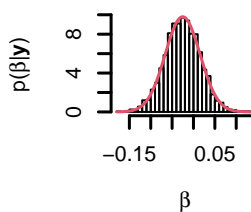**(p)**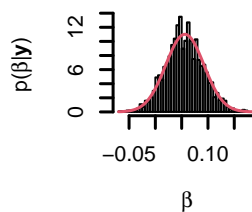

Supplement: Supplementary file 2 — Supporting Information [file BIMJ-63-289-s001.zip › Code_and_Data/Code_and_Data/figures/supp_figure2.pdf]

**(a)**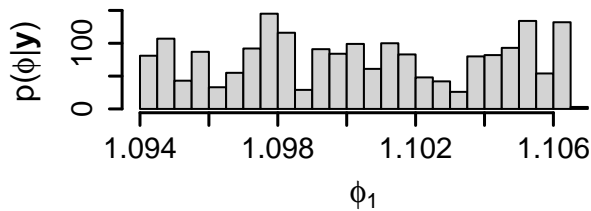**(b)**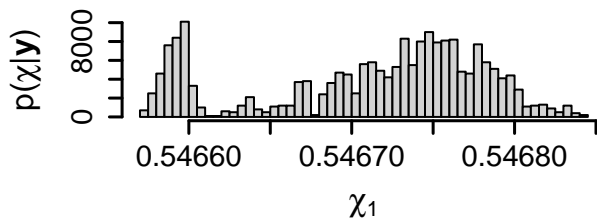**(c)**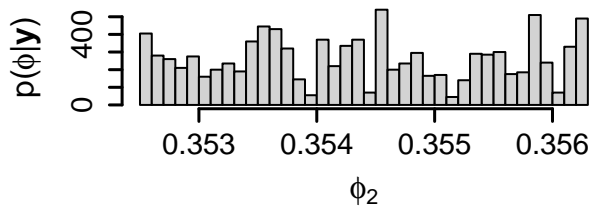**(d)**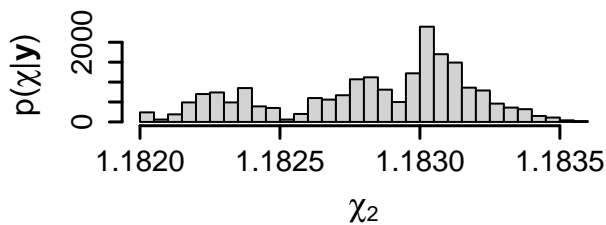**(e)**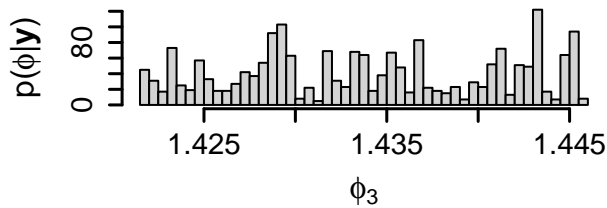**(f)**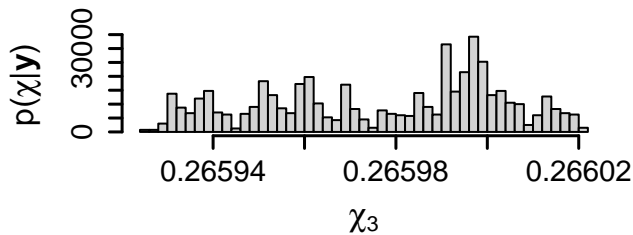**(g)**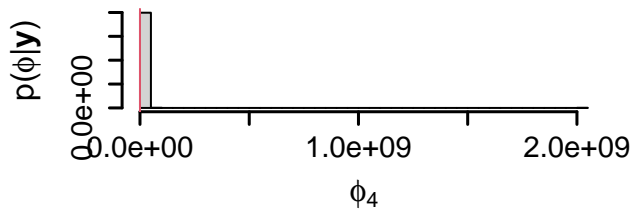**(h)**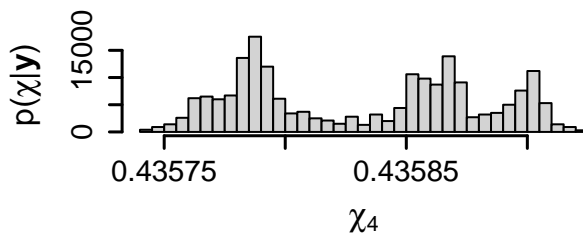

Supplement: Supplementary file 2 — Supporting Information [file BIMJ-63-289-s001.zip › Code_and_Data/Code_and_Data/figures/supp_figure3.pdf]

**(a)**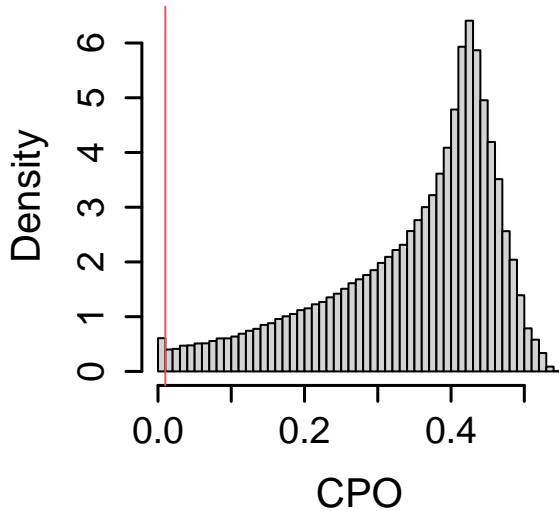**(b)**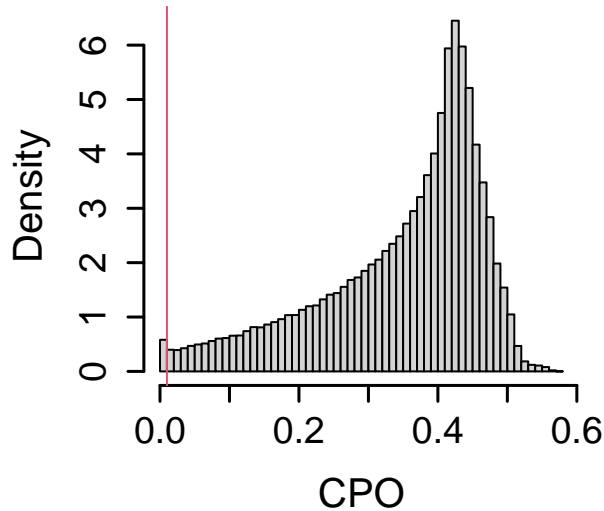**(c)**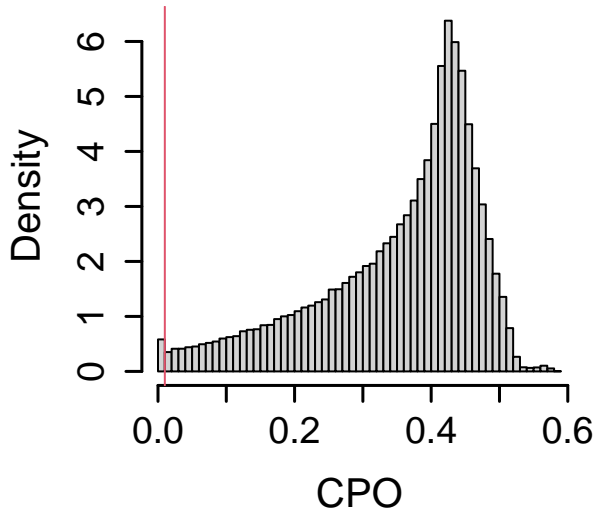**(d)**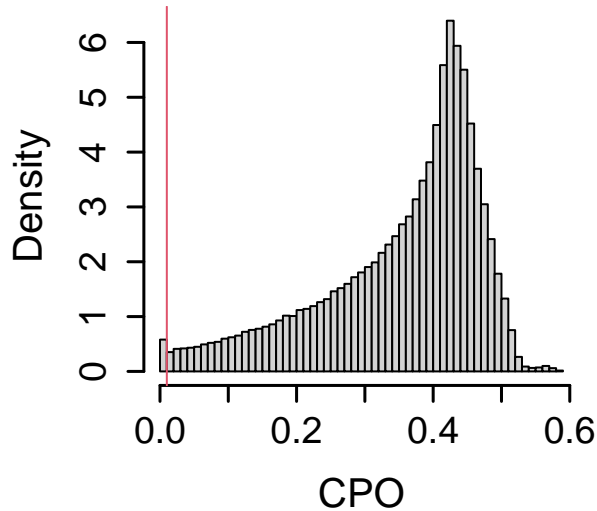

Supplement: Supplementary file 2 — Supporting Information [file BIMJ-63-289-s001.zip › Code_and_Data/Code_and_Data/figures/supp_figure4.pdf]
